# Lifestyle, Dietary Habits, and Modifiable Risk Factors in Patients with Acute Pulmonary Embolism: Protocol for a Prospective Observational Study with Development and Validation of a Composite Lifestyle and Nutrition Questionnaire

**DOI:** 10.3390/jcm15197573

**Published:** 2026-09-29

**Authors:** Carina Gib, Alina Anton, Andreea-Adriana Neamțu, Teodor-Andrei Maghiar, Raluca-Andreea Jupaneant, Laura Maghiar, Teodora Maria Bodog, Emanuel Ionuț Poplicean, Ion-Costel Epuras, Octavian Crețu, Daniel Florin Lighezan

**Affiliations:** 1 Doctoral School, Victor Babeș University of Medicine and Pharmacy Timișoara, Eftimie Murgu Square, No. 2, 300041 Timișoara, Romania; carina.gib@umft.ro (C.G.); andreea.jupaneant@umft.ro (R.-A.J.); emanuel.poplicean@umft.ro (E.I.P.); ion.epuras@umft.ro (I.-C.E.); 2 Center of Advanced Research in Cardiology and Hemostasology, Victor Babeș University of Medicine and Pharmacy Timișoara, Eftimie Murgu Square, No. 2, 300041 Timișoara, Romania; dlighezan@umft.ro; 3 Department of Toxicology, Victor Babeș University of Medicine and Pharmacy Timișoara, Eftimie Murgu Square, No. 2, 300041 Timișoara, Romania; dolghi.alina@umft.ro; 4 Research Centre for Pharmaco-Toxicological Evaluation, Victor Babeș University of Medicine and Pharmacy Timișoara, Eftimie Murgu Square, No. 2, 300041 Timișoara, Romania; 5 Department of Pathology, Clinical County Emergency Hospital of Arad, Andrenyi Karoly Str., No. 2–4, 310037 Arad, Romania; 6 Department of Pathology, “Pius Brinzeu” Clinical County Emergency Hospital Timișoara, Liviu Rebreanu Boulevard, No. 156, 300723 Timișoara, Romania; 7 Department of Surgical Disciplines, Faculty of Medicine and Pharmacy, University of Oradea, Universității Str., No. 1, 410087 Oradea, Romania; bodog.teodoramaria@student.uoradea.ro; 8 Department of Surgery, Pelican Hospital, Corneliu Coposu Str., No. 2, 410450 Oradea, Romania; 9 Department of Psycho-Neurosciences and Rehabilitation, Faculty of Medicine and Pharmacy, University of Oradea, Universității Str., No. 1, 410087 Oradea, Romania; laura.maghiar@uoradea.ro; 10 Department of Dermatovenerology, Clinical County Emergency Hospital Bihor, 410169 Oradea, Romania; 11 Bihor County Emergency Clinical Hospital, 410169 Oradea, Romania; 12 Doctoral School of Biomedical Sciences, University of Oradea, Universității Str., No. 1, 410087 Oradea, Romania; 13 Center of Research and Innovation in Personalized Medicine of Respiratory Diseases (CRIPMRD), Victor Babeș University of Medicine and Pharmacy Timișoara, Eftimie Murgu Square, No. 2, 300041 Timișoara, Romania; 14 Oftalmo Sensory-Tumor Research Center–ORL (EYE-ENT), Victor Babeș University of Medicine and Pharmacy Timișoara, Eftimie Murgu Square, No. 2, 300041 Timișoara, Romania; 15 Department of Surgery I—Clinic of Surgical Semiotics & Thoracic Surgery, Victor Babeș University of Medicine and Pharmacy Timișoara, Eftimie Murgu Square, No. 2, 300041 Timișoara, Romania; octavian.cretu@umft.ro; 16 Center for Hepato-Biliary and Pancreatic Surgery, Victor Babeș University of Medicine and Pharmacy Timișoara, Eftimie Murgu Square, No. 2, 300041 Timișoara, Romania; 17 Department of Internal Medicine I, Victor Babeș University of Medicine and Pharmacy Timișoara, Eftimie Murgu Square, No. 2, 300041 Timișoara, Romania

**Keywords:** pulmonary embolism, venous thromboembolism, risk stratification, Mediterranean diet, physical activity, smoking, laboratory biomarkers, questionnaire validation, cross-cultural adaptation, prospective cohort

## Abstract

**Background/Objectives**: Modifiable lifestyle factors are established determinants of venous thromboembolism risk, but their relationship with the severity and early course of acute pulmonary embolism (PE) is poorly characterized, and no validated Romanian-language instrument captures these domains in the acute PE setting. This protocol describes a prospective observational study with two distinct components: (i) a measurement component that develops and validates the Pulmonary Embolism Lifestyle and Nutrition Questionnaire (PE-LNQ), a composite patient-completed instrument, and (ii) a clinical component that analyzes the associations between lifestyle factors, routine clinical and laboratory data, and the severity and early outcomes of acute PE. **Methods**: Phase 1 comprises cross-cultural adaptation, content validation, piloting, and COSMIN-based psychometric evaluation. Its primary endpoints are test–retest reliability (ICC ≥ 0.70) and confirmation of ≥75% of a priori construct-validity hypotheses, including convergent validity against measured anthropometry and routine biomarkers. Phase 2 enrolls consecutive adults with CTPA-confirmed acute PE in a staged design (minimum 220 patients single-center; approximately 400 with a planned multicenter extension). The primary clinical endpoint is intermediate-/high-risk PE (vs. low risk) by sPESI/PESI, with the ordinal PESI class (I–V) as the key secondary endpoint. Severity data and a pre-specified panel of routine admission laboratory parameters are extracted from the medical record without study-specific sampling; patients are followed for in-hospital, 3-, and 6-month adverse events. The confirmatory analysis is multivariable logistic regression; ordinal, survival, incremental-value, and biomarker analyses are pre-specified as secondary or exploratory. **Expected Results**: The PE-LNQ is expected to show adequate reliability and validity, and less favorable lifestyle profiles to be associated with higher severity class and more early adverse events. **Conclusions**: This study will deliver a validated, freely reusable instrument and clarify the prognostic relevance of modifiable lifestyle factors, supporting their integration into risk assessment and secondary prevention.

## 1. Introduction

Acute pulmonary embolism (PE) is the third most frequent acute cardiovascular syndrome after myocardial infarction and stroke, and the most severe clinical manifestation of venous thromboembolism (VTE), a disease affecting an estimated 10 million people every year worldwide [1,2,3]. Despite advances in diagnosis and anticoagulation, PE remains an important cause of cardiovascular morbidity and mortality, with roughly 20% of patients with VTE dying within the first year [4]. Although age-standardized PE-related mortality declined in most high-income countries between 2000 and 2015, this decline has recently slowed or stalled, and unfavorable trends persist in younger adults and in Central and Eastern Europe [5,6].

Risk stratification underpins the contemporary management of acute PE [3,7]. The 2019 European Society of Cardiology (ESC) guidelines recommend an integrated approach combining hemodynamic status, validated clinical scores, imaging assessment of right ventricular (RV) dysfunction, and biomarkers of myocardial injury [3], an approach consolidated by the recently issued 2026 multi-society American guideline [8] and complemented by current antithrombotic therapy recommendations [9]. The Pulmonary Embolism Severity Index (PESI) and its simplified version (sPESI) identify low-risk patients eligible for outpatient management and those at higher risk of early death [10,11], and their prognostic performance has been confirmed in large contemporary cohorts [12]. Elevated cardiac troponin remains robustly associated with short-term mortality [13], and the right-to-left ventricular (RV/LV) diameter ratio measured on computed tomography provides incremental prognostic information [14]. Nevertheless, the discriminatory capacity of existing scores remains perfectible, and research is increasingly directed toward additional, reproducible, and accessible prognostic markers, including radiomics and machine-learning approaches applied to computed tomography pulmonary angiography (CTPA) [15,16].

Among the most accessible of such markers are routine admission laboratory parameters, which are obtained in virtually every hospitalized patient with PE at no additional cost. Plasma lactate above 2 mmol/L identifies normotensive patients at high risk of early adverse outcomes independently of RV dysfunction and troponin [17,18]; admission hyponatremia [19] and hyperglycemia [20] are independent predictors of 30-day mortality in large PE cohorts; impaired renal function marks acute kidney injury and predicts early complications [21]; and C-reactive protein (CRP) [22], low serum albumin [23], red cell distribution width (RDW) [24], and the neutrophil-to-lymphocyte and platelet-to-lymphocyte ratios (NLR, PLR) [25] have each been associated with RV dysfunction or mortality after acute PE. Several of these parameters—glucose, lipid profile, CRP, leukocyte and erythrocyte counts—are at the same time biological correlates of the lifestyle exposures of interest: traditional cardiometabolic risk factors cluster with VTE [26], smoking causally raises white and red blood cell counts [27], and adherence to a Mediterranean dietary pattern is related to adiposity and a cardiometabolic profile [28]. Routine laboratory data therefore offer, simultaneously, objective severity covariates, potential mediators, and independent anchors against which patient-reported lifestyle can be checked.

Modifiable risk factors are firmly established determinants of VTE occurrence. Obesity substantially increases VTE risk—approximately two-fold in prospective cohorts and more in some case–control series—through mechanical, inflammatory, and prothrombotic mechanisms [4,29], and physical inactivity and sedentary behavior are associated with incident VTE in meta-analyses of prospective cohorts, with a dose–response relationship reported for sedentary time [30,31]. Dietary pattern is an emerging factor: in two prospective Swedish cohorts, higher adherence to an anti-inflammatory diet was associated with an approximately 9% lower VTE risk, with a protective effect confined to smokers, suggesting interaction between diet and other modifiable factors [32]; higher ultra-processed food intake has been prospectively associated with greater VTE incidence [33], in line with the broad adverse cardiometabolic profile of ultra-processed foods [34]; and Mediterranean-type dietary patterns are consistently associated with lower cardiovascular risk in both primary and secondary prevention [35]. Mendelian randomization analyses support a causal effect of smoking on VTE [36], whereas the associations of alcohol [37] and caffeinated beverages [38] with VTE appear neutral or modest; electronic cigarettes, whose use is increasingly prevalent, have measurable adverse cardiovascular effects of as yet undefined thrombotic relevance [39]. Cancer remains the dominant acquired provoking factor for VTE [40].

Although these factors are well studied as determinants of VTE incidence, their relationship with the severity and early clinical course of an established acute PE episode remains insufficiently explored and is partly counterintuitive: several contemporary analyses describe an “obesity paradox”, with lower short-term mortality among patients with obesity hospitalized for PE [41,42], prompting calls for better phenotyping of body composition and lifestyle in this population [43]. Moreover, up to half of PE survivors develop persistent functional limitation (the “post-PE syndrome”), to which deconditioning, obesity, and physical inactivity are major contributors [44]; cardiopulmonary exercise testing documents reduced exercise capacity in a substantial proportion of patients after PE [45], and structured exercise interventions after PE appear safe and beneficial [46]. Current European prevention guidelines emphasize precisely these lifestyle domains [47], yet patients’ knowledge of VTE risk factors appears limited in the few surveys available [48]. Together, these observations make the systematic, standardized assessment of pre-event lifestyle a clinically meaningful component of PE evaluation—particularly in Romania, where cardiovascular mortality and the burden of modifiable risk factors are among the highest in Europe [49,50], and where dietary patterns are shaped by strong seasonal and traditional influences and recent disruptions [51].

Nutrition is, moreover, not only a pre-event exposure but also a component of care after the acute episode, for four reasons. First, nutritional status at presentation carries prognostic information: low serum albumin is associated with short-term poor prognosis [23], and the Controlling Nutritional Status (CONUT) score—computed from serum albumin, total cholesterol, and lymphocyte count—identified malnutrition in more than half of 308 consecutive patients hospitalized for acute PE and independently predicted in-hospital mortality [52]. Second, diet interacts with anticoagulant therapy, which every patient receives for at least three months: dietary vitamin K intake influences anticoagulation stability in patients treated with vitamin K antagonists, with current evidence favoring a consistent rather than a restricted intake [53], and in the RIETE registry (19,237 patients with VTE), low-density lipoprotein (LDL) cholesterol below 70 mg/dL was associated with a higher risk of bleeding during anticoagulation [54], suggesting that diet-sensitive lipid variables may bear on treatment safety as well as on cardiometabolic risk. Third, body weight—the most diet-dependent of the modifiable factors—is associated with the risk of recurrence after a first unprovoked VTE [55] and with the deconditioning that underlies post-PE functional limitation [44]. Fourth, the post-PE period is the natural window for secondary-prevention counseling on diet, weight, physical activity, and smoking, for which Mediterranean-type dietary patterns provide the best-supported framework [35,47]. Standardized measurement of nutrition-related behavior at the index episode is therefore a prerequisite for identifying the patients most likely to benefit from such counseling.

Studying these associations rigorously requires reliable measurement of the exposures. Internationally validated instruments exist for each lifestyle domain taken separately: the International Physical Activity Questionnaire–Short Form (IPAQ-SF) for physical activity [56,57], with recent re-validations in clinical and general populations [58,59]; the 14-item Mediterranean Diet Adherence Screener (MEDAS) for dietary pattern [28,60,61,62]; and the Fagerström Test for Nicotine Dependence (FTND) for smoking [63,64]. However, to our knowledge, no validated Romanian-language version of the MEDAS has been published, and no composite instrument integrates these lifestyle domains in a format tailored to patients with an acute PE episode. Combining previously validated scales into a new composite instrument, translating it into a new language, and administering it in a new clinical context all require formal evaluation of its measurement properties, following established recommendations for cross-cultural adaptation [65] and the COSMIN framework and reporting guidance for studies of measurement properties [66,67,68,69,70].

This study, therefore, has two components with distinct objectives, hypotheses, and endpoints. The measurement component (Phase 1) aims to develop the Pulmonary Embolism Lifestyle and Nutrition Questionnaire (PE-LNQ) and to evaluate its measurement properties—content validity, reliability, and construct validity, including convergent validity against objective clinical measurements—so that it may serve as a standardized, freely available tool for clinical practice and research in VTE. Its hypothesis is that the PE-LNQ has adequate measurement properties in patients with acute PE; its primary endpoints are test–retest reliability (ICC ≥ 0.70 for the principal continuous scores) and confirmation of at least 75% of the a priori construct-validity hypotheses (Section 2.5.4). The primary objective of the clinical component (Phase 2) is to analyze the association between modifiable lifestyle factors (obesity, physical activity, Mediterranean diet adherence, and smoking) and the severity of acute PE at presentation. Its primary hypothesis is that less favorable lifestyle profiles are associated with a higher probability of intermediate-/high-risk PE; its primary endpoint is the dichotomized sPESI/PESI risk class, with the ordinal PESI class (I–V) as the key secondary endpoint (Section 2.4.3). Secondary objectives are (i) to assess the association of these factors with early (in-hospital, 3- and 6-month) adverse outcomes; and (ii) to describe the modifiable risk-factor, nutritional, and cardiometabolic profile of a contemporary Romanian PE cohort. Exploratory objectives are to examine correlations between lifestyle indicators and biological and imaging severity markers, including a pre-specified panel of routine admission laboratory parameters, and to assess whether lifestyle factors add prognostic information beyond the sPESI and routine biomarkers.

## 2. Materials and Methods

### 2.1. Study Design and Setting

This is a prospective, observational cohort study initiated in the Clinical Department of Internal Medicine of the Municipal Emergency Clinical Hospital of Timișoara (Spitalul Clinic Municipal de Urgență Timișoara), Romania—a university-affiliated tertiary center—with a pre-specified staged extension to additional centers. Patients are enrolled consecutively at the index PE episode and followed prospectively. This study comprises two integrated phases: Phase 1, the development, cross-cultural adaptation, and validation of the PE-LNQ (Section 2.5); and Phase 2, the prospective cohort analysis of associations between lifestyle factors, routine clinical and laboratory data, and PE severity and early outcomes. All laboratory data are those generated by routine care; this study requires no blood samples or investigation beyond the questionnaire, a waist-circumference measurement, and the follow-up contacts. Reporting follows the STROBE statement for cohort studies [71] and the COSMIN taxonomy, quality criteria, and reporting guidance for the measurement-validation component [66,67,70].

Additional centers are selected according to pre-specified criteria intended to ensure geographical, demographic, and care-setting diversity: (i) public university or county emergency hospitals whose internal medicine, cardiology, or pulmonology departments manage at least 50 CTPA-confirmed PE episodes per year; (ii) 24/7 availability of CTPA and of the routine Tier 1–2 laboratory panel (Section 2.4.1); (iii) location in a historical region of Romania other than Banat (Transylvania, Crișana–Maramureș, Moldavia, Muntenia–Oltenia, or Dobruja), with at least one center serving a predominantly rural or mixed urban–rural catchment area; (iv) local ethics approval and investigators trained in the study procedures and questionnaire administration before site activation; and (v) capacity to perform the 3- and 6-month follow-up. No single center will contribute more than 40% of the multicenter enrollment, and the sociodemographic composition of the cohort (age, sex, area of residence, and educational level) will be monitored during enrollment against national population data.

### 2.2. Study Population and Eligibility Criteria

Inclusion criteria: age ≥ 18 years; a first or recurrent episode of acute PE objectively confirmed on computed tomography pulmonary angiography (CTPA); and provision of informed consent.

Exclusion criteria: inability to provide informed consent or complete the lifestyle questionnaire (e.g., severe cognitive impairment); PE diagnosed only incidentally without clinical suspicion, at the investigator’s discretion; and pregnancy-associated PE, which is analyzed separately. Patients with hemodynamic instability may be enrolled once stabilized, with the questionnaire completed according to the standardized timing and assistance rules described in Section 2.6.

The validation subsamples (expert panel excepted) are drawn from the same population. For test–retest administration, patients must be clinically stable at both administrations and must not have experienced a major intercurrent event (e.g., recurrence, decompensation, and escalation of therapy) between them.

### 2.3. Sample Size

Sample size planning addresses the two phases of this study separately: the observational cohort (primary and secondary clinical objectives) and the questionnaire validation.

Cohort phase. The primary analysis compares the probability of intermediate-/high-risk severity class between patients with and without an adverse lifestyle exposure. Sample sizes were computed for a two-sided α = 0.05 and 80% power using the normal-approximation comparison of two proportions with unequal allocation, assuming a 40% prevalence of intermediate-/high-risk class among unexposed patients—consistent with contemporary PE cohorts [12]—and exposure prevalences of 30–50%, as plausible for obesity, physical inactivity, low Mediterranean diet adherence, and smoking in the Romanian population [49,50,51]. Crude estimates were inflated by a design factor of 1.2 to account for the loss of precision from multivariable adjustment (variance inflation for correlated covariates) and then by 15% anticipated attrition. Table 1 presents the resulting scenario grid.

Based on this grid, enrollment proceeds in two pre-specified stages. The single-center first stage targets a minimum of 220 patients, which provides approximately 80% power to detect strong associations (OR ≥ 2.5) at exposure prevalences of 40–50% after adjustment and attrition (adjusted requirement 216–225), supports all validation-phase analyses, and yields stable estimates of the severity-class distribution. The planned multicenter extension targets approximately 400 patients in total, which provides 80% power for the principal scenario of a moderate association (OR = 2.0 at 40% exposure prevalence; adjusted requirement 390). A blinded reassessment of the severity-class distribution after the first 100 patients will verify the plausibility of the design assumptions without examining exposure–outcome associations.

The events-per-variable (EPV) budget is consistent with these targets: with 40–50% of patients in the intermediate-/high-risk class, the first stage yields approximately 75–95 “events” for the primary logistic model after attrition—supporting up to 8 degrees of freedom at EPV ≥ 10 (exposure plus the pre-specified covariates age, sex, active cancer, cardiopulmonary comorbidity, and provoked status)—and the multicenter extension approximately 135–170, permitting the pre-specified adjustment set with margin. Routine laboratory variables are deliberately kept out of the primary adjustment set and enter only secondary and sensitivity models (Section 2.7), so that the EPV budget of the primary analysis is preserved. Should a prognostic-model extension (re-estimation of a lifestyle-augmented risk score) be undertaken, the minimum sample size will be recalculated with modern criteria based on anticipated model performance rather than EPV alone [72].

For secondary time-to-event outcomes, assuming a 10–15% cumulative incidence of the adverse-event composite by 6 months, the first stage yields approximately 19–28 events—sufficient only for parsimonious Cox models (exposure plus one to two covariates at EPV ≥ 10)—and the multicenter extension approximately 34–51 events; these analyses are therefore considered supportive rather than confirmatory. Analyses treating the exposures continuously (per 1 standard deviation of MET-minutes/week; per MEDAS point) and the ordinal modeling of PESI classes I–V are expected to increase statistical efficiency relative to the dichotomized scenarios in Table 1, making the grid conservative.

Validation phase. Sample size targets follow established recommendations for studies of measurement properties [67,73,74]. Content validity requires at least eight experts, allowing computation of item-level content validity indices with acceptable precision [73]. Face-validity piloting includes 15 patients with cognitive debriefing. The psychometric analyses (internal consistency, construct validity, floor/ceiling effects) will be performed on the first 130 consecutive patients at minimum—exceeding the ≥100 threshold considered adequate in COSMIN-based quality criteria [67] and sufficient to detect correlations of |r| ≥ 0.25 with 80% power at α = 0.05. The test–retest subsample targets at least 80 clinically stable patients: by the method of Walter et al., 79 subjects with two administrations provide 80% power to demonstrate that an ICC of 0.70 differs from a null value of 0.50 [74]; this sample also bounds the 95% confidence interval half-width at approximately ±0.10–0.12 for an observed ICC of 0.75 and at approximately ±0.16 for a weighted kappa of 0.70. All validation targets are accommodated within the first-stage cohort enrollment.

### 2.4. Variables and Data Collection Instruments

Two complementary data sources are used: an investigator-completed clinical dataset extracted from the medical record and the patient-completed PE-LNQ.

#### 2.4.1. Routine Clinical and Laboratory Data

The clinical dataset comprises exclusively information generated during routine care of the index episode, extracted into a standardized case report form (Table 2). It covers demographics; measured anthropometry (height and weight recorded by nursing staff at admission as part of routine assessment, and waist circumference—the only study-added measurement—taken with a tape at the first stable assessment); vital signs and hemodynamic status at presentation; comorbidities summarized by the Charlson Comorbidity Index; chronic medication with particular attention to lipid-lowering, glucose-lowering, antihypertensive, antiplatelet, hormonal, and anticoagulant therapy; PESI and sPESI scores and risk class; provoked versus unprovoked status; CTPA findings including the RV/LV diameter ratio and, where available, the vascular obstruction index; treatment received (anticoagulant class, reperfusion therapy); length of stay; and discharge destination.

The laboratory panel is restricted to parameters that form part of the standard admission workup of patients with suspected or confirmed PE in the participating center(s); no study-specific sampling is performed. The first sample obtained at, or within 24 h of, PE diagnosis—and before reperfusion therapy where applicable—is used, and the time from symptom onset and from diagnosis to sampling is recorded. The panel is organized in three tiers according to its intended use. Tier 1 comprises established severity biomarkers: high-sensitivity cardiac troponin (I or T, interpreted against the assay-specific 99th percentile), natriuretic peptides (BNP or NT-proBNP), D-dimer, and lactate [3,13,17,18]. Because D-dimer concentrations rise physiologically with age, D-dimer is analyzed on the log scale with age in the model and, descriptively, classified against the age-adjusted threshold (age × 10 µg/L, fibrinogen-equivalent units, in patients older than 50 years), whose safety has been established in suspected PE and recently confirmed in suspected deep vein thrombosis [75,76,77]. Tier 2 comprises routine parameters with documented prognostic value in acute PE: serum sodium [19], glucose [20], creatinine with estimated glomerular filtration rate (eGFR) calculated with the 2021 CKD-EPI creatinine equation [21,78], albumin [23], CRP [22], and the complete blood count with hemoglobin, hematocrit, leukocyte differential, platelet count, and RDW, from which the NLR and PLR are derived [24,25]. Tier 3 comprises the cardiometabolic profile that links lifestyle to biology: total, LDL, and HDL cholesterol, triglycerides, uric acid, and glycated hemoglobin (HbA1c) where measured, together with fasting status at sampling. Beyond their role as cardiometabolic anchors, lipid parameters have been associated with clinically relevant outcomes in VTE—notably, LDL cholesterol < 70 mg/dL (1.8 mmol/L) with a higher bleeding risk during anticoagulation [54]—and are therefore also examined in relation to bleeding during follow-up (Section 2.4.3). The CONUT nutritional-status score (serum albumin, total cholesterol, and total lymphocyte count) is derived from the same sample [52]. From Tiers 2–3 laboratory parameters (glucose, lipid profile), vital signs, and anthropometry, the components of the metabolic syndrome are derived according to the harmonized 2009 definition (elevated waist circumference, triglycerides ≥ 1.7 mmol/L, reduced HDL cholesterol, blood pressure ≥ 130/85 mmHg or treatment, and fasting glucose ≥ 5.6 mmol/L or treatment) [79]. All values are recorded in SI units with the analyzer and assay used; in the multicenter extension, assay-dependent parameters (troponin, natriuretic peptides, D-dimer) are analyzed as site-specific standardized values or dichotomized at site-specific cut-offs.

#### 2.4.2. Patient-Reported Lifestyle and Risk Factors (PE-LNQ)

Habitual pre-event lifestyle over the approximately 12 months preceding the episode is captured with the PE-LNQ, a structured composite questionnaire organized in eight sections: (A) sociodemographic and anthropometric data, including occupation and self-reported pre-event height and weight for BMI and recent weight change; (B) personal and family VTE history, provoking factors, and comorbidities; (C) the IPAQ-SF (MET-minutes/week; low/moderate/high categories) [56]; (D) the 14-item MEDAS (0–14; ≥9 = good adherence) [28,60]; (E) complementary dietary-habit items (meal pattern, breakfast regularity, hydration, coffee, ultra-processed food frequency, and dietary supplements); (F) smoking status, pack-years, electronic-cigarette/heated-tobacco use, and the FTND (0–10) for current smokers [63]; (G) alcohol intake (quantity–frequency); and (H) patient perception of the triggering event and intentions to change. The structure, item sources, and scoring outputs are summarized in Table 3; the full instrument is provided as Supplementary Materials (Questionnaire S1: Romanian version; Questionnaire S2: English translation).

#### 2.4.3. Outcomes

The primary outcome is PE severity class (low vs. intermediate/high risk per sPESI/PESI). This dichotomy mirrors the principal management decision in current guidelines—eligibility for early discharge or home treatment versus hospital-based care and monitoring [3,8]—and separates patients with markedly different early mortality (approximately 1% vs. 11% at 30 days in the sPESI derivation cohort [11]). Because intermediate- and high-risk PE are clinically heterogeneous and high-risk patients are expected to be few, the ordinal PESI class (I–V) is designated the key secondary endpoint (Section 2.7.2); in sensitivity analyses, the four-level 2019 ESC classification (low, intermediate-low, intermediate-high, and high risk) is modeled as an ordinal outcome, and the primary analysis is repeated after exclusion of high-risk patients. Secondary outcomes are a composite of early adverse events (all-cause death, PE-related death, recurrent VTE, and clinical deterioration requiring escalation of therapy) assessed in-hospital and at 3 and 6 months, and the continuous severity markers listed in Table 2 (RV/LV ratio and Tier 1–2 laboratory parameters). Bleeding events during anticoagulation are recorded as a separate safety outcome, classified as major bleeding according to the International Society on Thrombosis and Haemostasis (ISTH) criteria [80], with clinically relevant non-major bleeding also recorded.

### 2.5. Questionnaire Development, Cross-Cultural Adaptation, and Validation

#### 2.5.1. Instrument Assembly and Translation

The PE-LNQ assembles three internationally validated instruments (IPAQ-SF [56,57], MEDAS [60,61], FTND [63]) with original items developed by the research team to cover domains relevant to VTE but not addressed by the constituent scales (provoking factors, complementary dietary habits, alcohol quantity–frequency, and patient perception). The Romanian versions of the constituent scales are produced by forward–backward translation in accordance with established guidelines for cross-cultural adaptation [65]: two independent forward translations by native Romanian speakers fluent in English (one with and one without a medical background); synthesis into a single version; back-translation by two independent bilingual translators blinded to the originals; and review by an expert committee resolving semantic, idiomatic, experiential, and conceptual discrepancies to produce the pre-final version. The content of validated-scale items is not altered; only the translation is adjusted.

#### 2.5.2. Content Validity

The pre-final version is evaluated by a panel of at least eight experts (internal medicine/cardiology, pulmonology, clinical nutrition, epidemiology/public health, and clinical research methodology), who rate the relevance and clarity of each item for the construct “pre-event lifestyle and nutrition of patients with acute PE” on a 4-point scale, following the content validity index methodology of Polit and Beck [73] within the COSMIN framework for content validity [68]. The item-level content validity index (I-CVI) and the average scale-level index (S-CVI/Ave) are computed: items with I-CVI ≥ 0.78 are retained, items with I-CVI 0.70–0.77 are revised, and original items with I-CVI < 0.70 are considered for removal (items belonging to validated scales are retained, with rewording confined to the translation level). An S-CVI/Ave ≥ 0.90 is targeted for the overall instrument.

#### 2.5.3. Face Validity, Pilot Testing, and Acceptability

The content-validated version is piloted in 15 patients with acute PE using cognitive debriefing (“think-aloud”) interviews addressing comprehension, ambiguity, recall burden, and cultural appropriateness; completion time and the need for assistance are recorded. Acceptability criteria are a mean completion time ≤ 20 min and <5% missing items; problematic items are reworded and re-piloted if necessary.

#### 2.5.4. Psychometric Evaluation

Measurement properties are evaluated following the COSMIN taxonomy [66] and the quality criteria of Terwee et al. [67] in at least the first 130 consecutive patients (Section 2.3). Internal consistency is estimated for the FTND; because the MEDAS is a formative index rather than a reflective scale, its internal consistency is reported descriptively, and reproducibility is emphasized at item level. Test–retest reliability is assessed by re-administering the PE-LNQ after 7–14 days to at least 80 clinically stable patients (Section 2.3). The statistical criteria for reliability, agreement, and floor/ceiling effects are detailed in Section 2.7.6. Construct validity is tested against a priori hypotheses, with ≥75% confirmation considered supportive [67]. The hypotheses deliberately use objective clinical and laboratory measurements as external anchors wherever possible: (i) IPAQ-SF activity is inversely associated with measured BMI and with daily sitting time (expected |r| 0.20–0.40); (ii) the MEDAS score is inversely associated with measured BMI, waist circumference, and triglycerides, and positively with HDL cholesterol (expected |r| 0.10–0.30, as reported in the PREDIMED validation cohort [28]); (iii) among current smokers, the FTND score correlates positively with cigarettes/day and pack-years, and smoking status is associated with higher hemoglobin, hematocrit, and leukocyte counts, consistent with the causal hematological effects of smoking [27]; (iv) patients with predominantly physical occupations attain higher IPAQ-SF categories than those with sedentary occupations (known-groups validity); and (v) self-reported pre-event weight and height (PE-LNQ Section A) agree with measured admission values (ICC ≥ 0.90 for BMI; mean bias within ±1 kg/m^2^), given the well-described tendency toward underestimation of self-reported BMI [81]. Because the acute-phase response lowers cholesterol fractions and raises triglycerides, lipid-based hypotheses are tested with adjustment for the time from symptom onset to sampling and for lipid-lowering therapy, with a sensitivity analysis restricted to patients not receiving such therapy [82]. Albumin-based analyses are likewise adjusted for CRP as a marker of the acute-phase response.

### 2.6. Study Procedures and Follow-Up

At inclusion, eligible patients receive the participant information sheet, provide written informed consent (Supplementary Materials, Consent Form S3), and complete the PE-LNQ referring to habitual behavior in the ~12 months preceding the episode; clinical and laboratory data are transcribed from the medical record into a standardized case report form, with the laboratory panel retrieved directly from the hospital laboratory information system to avoid transcription error. In the test–retest subsample, the questionnaire is re-administered 7–14 days after the first completion—during hospitalization or at the first outpatient contact—under the same mode of administration, provided the patient remains clinically stable. Follow-up is performed at hospital discharge and at 3 and 6 months through scheduled visits or structured telephone contact, recording vital status, recurrence, and clinical events. A standardized data-collection protocol and source-data verification are used to minimize missing or inconsistent data.

Questionnaire timing and assistance are standardized to limit recall and administration bias. The PE-LNQ is administered once the patient is hemodynamically stable (no vasopressor or inotropic support and no need for high-flow oxygen or ventilatory support), preferably within the first 72 h of admission and in all cases before discharge; the time from admission to administration is recorded. Administration follows a written script by trained investigators, with a standardized recall anchor (“the 12 months before your first symptoms”) supported by calendar landmarks (seasons and major holidays). Patients complete the questionnaire themselves whenever possible; interviewer assistance—reading items aloud and recording answers verbatim, without prompting—is offered to patients with visual, literacy, or fatigue-related limitations. A proxy (a cohabiting relative familiar with the patient’s habits) is used only when the patient cannot respond reliably, and only for the factual Sections A–G, never for Section H. The mode of administration (self-completed, interviewer-assisted, or proxy) is recorded for every questionnaire; proxy-completed questionnaires are excluded from the psychometric and test–retest analyses, and the mode of administration and the time from admission to administration are examined in sensitivity analyses of the clinical component.

### 2.7. Statistical Analysis

#### 2.7.1. General Principles

Analyses will be performed in R (version ≥ 4.3; R Foundation for Statistical Computing, Vienna, Austria; packages pROC, psych, irr, mice, survival, and rms) and/or IBM SPSS Statistics (version ≥ 28; IBM Corp., Armonk, NY, USA), with two-sided α = 0.05. The distribution of continuous variables will be assessed with the Shapiro–Wilk test and quantile–quantile plots; results will be summarized as mean ± standard deviation or median (interquartile range), and categorical variables as counts and percentages. Right-skewed laboratory variables (troponin, natriuretic peptides, D-dimer, CRP, NLR, PLR, triglycerides) will be log-transformed for parametric analyses. Effect estimates will be reported with 95% confidence intervals throughout, and interpretation will rest on estimation rather than on statistical significance alone. For the family of exploratory correlation analyses, the false discovery rate will be controlled with the Benjamini–Hochberg procedure. No formal interim efficacy analyses are planned.

Analyses follow a pre-specified hierarchy that is maintained throughout this protocol and will be retained in all resulting reports. Confirmatory analyses are limited to the primary analysis of the clinical component (Section 2.7.2) and the pre-specified reliability and construct-validity criteria of the measurement component (Section 2.7.6). The ordinal PESI analysis is the key secondary analysis, and the time-to-event analyses are supportive, given the limited number of expected events (Section 2.3). All other analyses—biomarker correlations, incremental prognostic value, dose–response, interaction, mediation, CONUT, and lipid–bleeding analyses—are exploratory and hypothesis-generating; they will be reported as such, with effect estimates and confidence intervals and without claims of confirmation, and, in the single-center stage, primarily descriptively.

#### 2.7.2. Primary Analysis

Baseline characteristics will be summarized by severity class, with between-group comparisons by the *t*-test or Mann–Whitney U test (continuous variables) and χ^2^ or Fisher’s exact test (categorical variables). The primary analysis is a multivariable binary logistic regression modeling intermediate-/high-risk severity class as a function of each modifiable lifestyle factor, entered both categorically (e.g., IPAQ-SF category; MEDAS ≥ 9 vs. <9; obesity, defined as measured admission BMI ≥ 30 kg/m^2^, with self-reported pre-event BMI used in a sensitivity analysis; current smoking) and continuously (per 1 standard deviation of MET-minutes/week; per MEDAS point; per 10 pack-years), adjusting for the pre-specified covariates age, sex, active cancer, cardiopulmonary comorbidity, and provoked status. Multicollinearity will be checked with variance inflation factors (VIF < 2.5). Model calibration will be assessed with the Hosmer–Lemeshow test and calibration plots, and discrimination with the area under the ROC curve; internal validation will use bootstrap resampling (1000 replicates) with optimism-corrected performance estimates [83]. As the key secondary analysis, a proportional-odds ordinal regression across PESI classes I–V will be fitted, with the proportional-odds assumption verified (Brant test) and a partial proportional-odds model used if violated. As a pre-specified secondary exposure, the metabolic syndrome (harmonized definition [79]) and the number of its components (0–5) will be analyzed in the same modeling framework. In the multicenter extension, between-center heterogeneity will be accounted for with a random intercept for center (mixed-effects models) or cluster-robust standard errors.

#### 2.7.3. Secondary Outcomes and Incremental Prognostic Value

The early adverse-outcome composite will be analyzed as a binary endpoint (in-hospital) and as a time-to-event endpoint (3 and 6 months) with Kaplan–Meier curves and Cox proportional hazards models, with a competing-risk (Fine–Gray) sensitivity analysis for recurrent VTE. The incremental prognostic value of lifestyle factors over the sPESI, alone and combined with troponin and lactate, will be explored as the change in area under the ROC curve, with reclassification metrics interpreted cautiously. Absolute risks and risk differences will accompany all relative measures. Full model specifications are provided in Supplementary Methods S6.

#### 2.7.4. Exploratory Biomarker, Dose–Response, and Interaction Analyses

Correlations between lifestyle indicators and continuous severity markers and Tier 2–3 laboratory parameters will be estimated as partial correlations adjusted for age and sex, with false discovery rate control. Sensitivity models will add routine markers (sodium, eGFR, glucose, albumin, and CRP) to the primary model; non-linear dose–response will be explored with restricted cubic splines; a multiplicative interaction between Mediterranean diet adherence and smoking will be tested, motivated by prior evidence of effect modification [32]; and mediation through cardiometabolic and inflammatory markers will be explored. The associations of the CONUT score with severity and outcomes, and of LDL cholesterol (<70 vs. ≥70 mg/dL) with bleeding during follow-up [52,54], will be examined in the same framework. Given the expected sample size, these analyses are hypothesis-generating and will be reported irrespective of statistical significance; full specifications are provided in Supplementary Methods S6.

#### 2.7.5. Missing Data

The extent and pattern of missing data will be reported for every variable, distinguishing laboratory parameters not measured as part of routine care (structurally missing, reported as such) from values missing for other reasons. Scale scores will be computed only when at least 13 of 14 MEDAS items, all 6 FTND items, and complete IPAQ-SF activity fields are available; single missing MEDAS items are scored 0 (conservative). The primary analyses use complete cases; as sensitivity analyses, multiple imputation by chained equations will be performed (m = 20 imputations, including outcomes, exposures, covariates, laboratory parameters, and auxiliary variables in the imputation model, with Rubin’s rules for pooling), under the missing-at-random assumption [84]. Divergence between complete-case and imputed estimates will be discussed explicitly.

#### 2.7.6. Validation-Phase Statistics

Content validity will be quantified by the item-level content validity index (I-CVI) and the average scale-level index (S-CVI/Ave) [73]. Internal consistency of the FTND will be estimated with Cronbach’s alpha, with values ≥0.70 considered adequate; because the MEDAS is a formative index, its alpha will be reported descriptively only. Test–retest reliability will be quantified by intraclass correlation coefficients (two-way random-effects model, absolute agreement, single measurement, ICC(2,1)) with 95% confidence intervals for continuous scores (MET-minutes/week, MEDAS total, FTND total) [85], by quadratic-weighted kappa for ordinal items, and by Cohen’s kappa for categorical items (κ ≥ 0.60 indicating substantial agreement); the standard error of measurement and the minimal detectable change at the 95% level (MDC95) will be derived from the ICC. Agreement for MET-minutes/week, and between self-reported and measured BMI, will additionally be examined with Bland–Altman plots and 95% limits of agreement, reported according to current standards [86]. Construct validity will be tested against the a priori hypotheses defined in Section 2.5.4, with ≥75% confirmation considered supportive [67]. Floor and ceiling effects will be considered present if more than 15% of respondents achieve the minimum or maximum score of a scale. Measurement stability across subgroups (age < 65 vs. ≥ 65 years; sex; self-completed vs. interviewer-assisted administration) will be examined descriptively.

#### 2.7.7. Reporting

The cohort analyses will be reported according to the STROBE statement [71] and the validation analyses according to the COSMIN reporting guideline for studies on measurement properties [70]. All pre-specified analyses are identified as such; any post hoc analysis will be labeled explicitly. A completed STROBE checklist for the present protocol, mapping each item to the section in which it is addressed, is provided as Table S4, and the corresponding mapping of the COSMIN reporting domains for the validation phase as Table S5, and the detailed specification of secondary and exploratory analyses as Supplementary Methods S6 (Supplementary Materials).

### 2.8. Data Management and Bias

Data are stored pseudonymously in a secure, access-controlled database, with range and consistency checks at entry and a 10% random sample double-checked against source documents. Consecutive enrollment limits selection bias. Recall bias in pre-event lifestyle reporting is acknowledged and mitigated by structured, validated instruments, by standardized questionnaire timing, recall anchors, and administration rules (Section 2.6), and by the objective anchors built into the validation hypotheses (measured anthropometry, hematological markers of smoking). Information bias in laboratory data is limited by using the first routine sample, by recording sampling time and fasting status, and by retrieving values directly from the laboratory information system; the influence of the acute-phase response on lipid fractions and albumin [82] and of lipid-lowering and glucose-lowering therapy is addressed by adjustment and pre-specified sensitivity analyses. Residual confounding is addressed through multivariable adjustment and pre-specified sensitivity analyses.

### 2.9. Ethical Considerations and Registration

This study will be conducted in accordance with the Declaration of Helsinki and has been approved by the Institutional Review Board of the Municipal Emergency Clinical Hospital of Timișoara (approval No. E-3780 of 18 August 2026). Written informed consent is obtained from every participant before any study procedure, using a participant information sheet and consent form approved together with the protocol (Supplementary Materials, Consent Form S3: Romanian version with English translation); the form explains the purpose of this study, the procedures (questionnaire, optional retest, waist measurement, use of routine clinical and laboratory data, and follow-up contacts), the voluntary nature of participation, the right to withdraw at any time without any consequence for medical care, and the pseudonymized handling of data. Where a patient cannot complete the questionnaire unaided, consent is still obtained from the patient, with a legally authorized representative or witness co-signing as applicable. Because the clinical and laboratory data are those generated by routine care, participation entails no additional blood sampling or investigation; the only burdens are questionnaire completion, a waist-circumference measurement, and follow-up contacts. Data are processed in compliance with Regulation (EU) 2016/679 (GDPR). Although registration is not mandatory for purely observational studies, this study will be registered in a public registry (ClinicalTrials.gov) before enrollment of the first participant, and any substantial protocol amendments will be documented and reported.

## 3. Expected Results

We anticipate enrolling at least 220 patients with confirmed acute PE in the first stage, and approximately 400 with the multicenter extension, and characterizing their modifiable risk factor and cardiometabolic profile. We expect that obesity, physical inactivity, low Mediterranean diet adherence, and current smoking will be associated with a higher-risk severity class at presentation and with a higher rate of early adverse outcomes, and that lifestyle indicators will correlate with biological and imaging markers of right ventricular strain and with routine inflammatory and metabolic parameters. Exploratory analyses will examine whether part of the lifestyle–severity association is transmitted through measurable cardiometabolic and inflammatory markers and whether lifestyle information adds prognostic value beyond the sPESI and routine biomarkers; these findings will be interpreted as hypothesis-generating. These associations, if confirmed, would support the prognostic relevance of modifiable factors beyond their established role in VTE incidence; conversely, a confirmed inverse (paradoxical) association for body mass index would contribute contemporary, prospectively collected evidence with measured anthropometry to the ongoing debate on the obesity paradox in PE [41,43].

With respect to the measurement objective, we expect the PE-LNQ to demonstrate satisfactory content validity (S-CVI/Ave ≥ 0.90), acceptable reliability (ICC ≥ 0.70 for the principal scores and κ ≥ 0.60 for categorical items), and confirmation of most construct-validity hypotheses, including agreement between self-reported and measured anthropometry and the expected relationships with lipid and hematological parameters, supporting its use as a standardized instrument for lifestyle and nutrition assessment in patients with VTE. The validated Romanian version and its English translation will be made freely available as Supplementary Materials of the resulting publications, together with their scoring rules, enabling reuse, clinical audit, and further validation by other groups and in other settings.

## 4. Discussion

This study will translate literature observations on diet, obesity, and physical activity in VTE into a contemporary Romanian clinical PE cohort, linking habitual lifestyle to objective severity, routine biomarkers, and short-term prognosis. Its strengths include prospective consecutive enrollment, objective PE confirmation, validated lifestyle instruments embedded in a formally validated composite questionnaire, and a pre-specified, staged sample size framework with an extended statistical analysis plan covering calibration, internal validation, competing risks, incremental prognostic value, dose–response modeling, and missing data handling. The systematic integration of routine clinical and laboratory data adds three distinct benefits at no additional cost or burden: objective severity covariates and inexpensive prognostic markers whose value is documented in PE [17,19,20,21,22,23,24,25]; biological anchors—measured anthropometry, lipid profile, hematological indices—against which the convergent validity of a self-report instrument can be tested more convincingly than by questionnaire-to-questionnaire comparison alone; and the possibility of exploring whether lifestyle acts on PE severity through measurable cardiometabolic and inflammatory pathways. To our knowledge, the PE-LNQ will be the first Romanian-language composite instrument for lifestyle and nutrition assessment validated in patients with acute PE; making the instrument and its scoring rules publicly available addresses a practical gap for clinical audit, secondary-prevention counseling, and multicenter research, and the a priori publication of this protocol together with its validation plan strengthens transparency and reproducibility.

By quantifying pre-event lifestyle with a validated instrument rather than chart-derived proxies, this study will also contribute data relevant to two active debates: the “obesity paradox” reported in hospitalized PE cohorts [41,42,43], and the role of pre-event physical activity and fitness in the risk of post-PE functional impairment, for which exercise-based interventions are emerging [44,45,46]. Standardized lifestyle assessment at the index episode may additionally furnish a practical anchor for individualized secondary-prevention counseling, in line with current prevention guidelines [47] and with the growing recognition that patient awareness of VTE risk factors is limited [48]. Nutritional assessment at the index episode is also directly relevant to the period of anticoagulation, during which nutritional status, dietary vitamin K intake, and lipid-related variables may bear on prognosis and treatment safety [52,53,54].

Anticipated limitations include the observational design; potential recall bias in pre-event lifestyle reporting, mitigated but not eliminated by standardized timing, recall anchors, and administration rules; the single-center setting of the first stage, addressed through the pre-specified multicenter extension whose sample size requirements are transparently laid out in Table 1; the reliance of the two-group power scenarios on assumed severity class and exposure prevalences, mitigated by the blinded design-assumption reassessment and by the greater efficiency of the continuous and ordinal analyses; the clinical heterogeneity of the combined intermediate-/high-risk category, addressed by the ordinal and four-level ESC analyses; the number of secondary and exploratory analyses relative to the sample size, which is why only the primary analyses are considered confirmatory; the validation of the PE-LNQ in a single clinical population, which may limit the generalizability of its measurement properties to other settings; and the formative nature of the MEDAS, which constrains the interpretation of classical internal consistency statistics. Laboratory data obtained during an acute illness carry their own caveats: lipid fractions and albumin are altered by the acute-phase response [82], HbA1c and lipid profile may not be measured in every patient, and assays differ between centers; these are addressed by adjusting lipid-based analyses for the time from symptom onset to sampling and for lipid-lowering therapy, by reporting structural missingness explicitly, and by site-specific standardization in the multicenter extension, but residual measurement heterogeneity cannot be excluded. In addition, AI-assisted imaging analysis (pursued in a parallel arm of the doctoral program) may refine the imaging severity markers used here [15,16]. This study constitutes the clinical component (Direction 2) of a broader doctoral research program on prognostic elements in PE.

## 5. Conclusions

This protocol defines a prospective observational study that couples the development and validation of a composite lifestyle and nutrition questionnaire (PE-LNQ) with the analysis of whether modifiable lifestyle factors are associated with the severity and early clinical course of acute PE, supported by a transparent, scenario-based sample size framework, an extended, pre-specified statistical analysis plan, and the systematic use of routine clinical and laboratory data as objective covariates and validation anchors. By integrating validated lifestyle instruments with standard clinical risk stratification and routinely available biomarkers—and by delivering a validated, freely reusable measurement tool—this study may identify accessible, actionable prognostic elements and inform secondary-prevention strategies in patients with PE.

## Figures and Tables

**Table 1:** Total sample size scenarios for the primary analysis (two-sided α = 0.05; power 80%; prevalence of intermediate-/high-risk severity class among unexposed patients 40%). Values are crude estimates, with estimates adjusted for multivariable analysis (design factor 1.2) and 15% attrition in parentheses.

Assumed Odds Ratio	Exposure Prevalence 30%	Exposure Prevalence 40%	Exposure Prevalence 50%
1.8	436 (616)	383 (541)	368 (520)
2.0	314 (444)	276 (390)	265 (375)
2.5	181 (256)	159 (225)	153 (216)

**Table 2:** Routine clinical and laboratory variables extracted from the medical record, with timing and intended analytical use.

Domain	Variables	Timing/Source	Analytical Use
Demographics and anthropometry	Age, sex, measured height, weight, and waist circumference; BMI	Admission (nursing record); waist at first stable assessment	Covariates; measured BMI as convergent-validity anchor for PE-LNQ Section A; metabolic-syndrome component (waist)
Hemodynamics and vital signs	Systolic/diastolic blood pressure, heart rate, respiratory rate, SpO_2_, and temperature	Presentation	PESI/sPESI computation; metabolic-syndrome component (blood pressure)
Comorbidity and medication	Charlson Comorbidity Index; active cancer; cardiopulmonary disease; lipid-lowering, glucose-lowering, antihypertensive, antiplatelet, hormonal, and anticoagulant therapy	Admission history and medication reconciliation	Pre-specified covariates; effect modifiers; interpretation of lipid and glucose values
Severity and imaging	PESI, sPESI class; CTPA RV/LV ratio; vascular obstruction index (where available); provoked/unprovoked status	Diagnosis	Primary outcome; continuous severity markers
Tier 1 laboratory: severity biomarkers	hs-troponin I/T, BNP/NT-proBNP, D-dimer, lactate	First routine sample ≤ 24 h from diagnosis, before reperfusion	Secondary outcomes; incremental-value reference model
Tier 2 laboratory: routine prognostic parameters	Sodium, glucose, creatinine and eGFR (CKD-EPI 2021), albumin, and CRP; complete blood count (hemoglobin, hematocrit, leukocytes and differential, platelets, and RDW); derived NLR and PLR	Same sample	Secondary continuous severity outcomes; correlations with lifestyle; sensitivity adjustment; exploratory mediation
Tier 3 laboratory: cardiometabolic profile	Total, LDL, and HDL cholesterol; triglycerides; uric acid; HbA1c (where measured); fasting status	Same sample	Convergent-validity anchors for MEDAS; metabolic-syndrome components; CONUT score; LDL cholesterol in exploratory bleeding analysis
Treatment and course	Anticoagulant class, reperfusion therapy, length of stay, and discharge destination	Hospitalization	Descriptive; covariates for outcome analyses

**Table 3:** Structure of the Pulmonary Embolism Lifestyle and Nutrition Questionnaire (PE-LNQ), version 2.0 (63 items).

Section	Domain	Items (*n*)	Source	Scoring/Output
A	Sociodemographic and anthropometric data	8	Original items	Descriptive; BMI (kg/m^2^); weight-change category
B	Medical history and VTE risk factors	10	Original items	Categorical risk-factor profile
C	Physical activity	7	IPAQ-SF	MET-minutes/week; low/moderate/high category
D	Mediterranean diet adherence	14	MEDAS	Total score 0–14; ≥9 = good adherence
E	Complementary dietary habits	6	Original items	Categorical indicators (meal pattern, breakfast, hydration, coffee, ultra-processed food, supplements)
F	Smoking and nicotine dependence	11	Original items + FTND	Smoking status; pack-years; e-cigarette use; FTND total 0–10
G	Alcohol intake	3	Original items	Frequency category; units/week
H	Patient perception and intentions	4	Original items	Categorical; free text

## Data Availability

The datasets generated during this study will be available from the corresponding author on reasonable request, subject to ethical and data-protection constraints. The PE-LNQ instrument (Supplementary Materials Questionnaires S1 and S2) may be used freely for non-commercial clinical and research purposes with citation of this protocol.

## Supplementary Materials

The following supporting information can be downloaded at: https://www.mdpi.com/article/10.3390/jcm15197573/s1, Questionnaire S1: PE-LNQ, version 2.0, Romanian version (patient-administered); Questionnaire S2: PE-LNQ, version 2.0, English translation; Consent Form S3: participant information sheet and informed consent form (Romanian version and English translation); Table S4: STROBE Statement checklist for reports of cohort studies, completed for the present protocol; Table S5: COSMIN reporting guideline for studies on measurement properties, mapped to the sections of the present protocol; Supplementary Methods S6: Detailed specification of secondary and exploratory analyses.
