# Supplementary material for: Lifestyle, Dietary Habits, and Modifiable Risk Factors in Patients with Acute Pulmonary Embolism: Protocol for a Prospective Observational Study with Development and Validation of a Composite Lifestyle and Nutrition Questionnaire
Source: J Clin Med. 2026 Sep 29;15(19):7573. doi: 10.3390/jcm15197573 (PMC13666219; doi:10.3390/jcm15197573)
Supplement: Supplementary file 1 [file jcm-15-07573-s001.zip › jcm-4559202-supplementary.pdf]

Supplementary Materials

Lifestyle, Dietary Habits, and Modifiable Risk Factors in Patients with Acute Pulmonary Embolism: Protocol for a Prospective Observational Study with Development and Validation of a Composite Lifestyle and Nutrition Questionnaire

Gib C., Anton A., Neamțu A.-A., Maghiar T.-A., Jupaneant R.-A., Maghiar L., Bodog T.M., Poplicean E.I., Epuras I.-C., Crețu O., Lighezan D.F.

Journal of Clinical Medicine — manuscript jcm-4559202 (Study Protocol), revised version

Contents

Questionnaire S1. Pulmonary Embolism Lifestyle and Nutrition Questionnaire (PE-LNQ), version 2.0 — Romanian version (patient-administered)

Questionnaire S2. PE-LNQ, version 2.0 — English translation

Consent Form S3. Participant information sheet and informed consent form (Romanian version with English translation)

Table S4. STROBE Statement checklist for reports of cohort studies, completed for the present protocol

Table S5. COSMIN reporting guideline for studies on measurement properties (version 2.0), mapped to the sections of the present protocol

Supplementary Methods S6. Detailed specification of secondary and exploratory analyses

Material suplimentar S1 — versiunea pre-finală în limba română (administrată pacientului), supusă validării de conținut și testării pilot descrise în protocol

Chestionarul privind Stilul de Viață și Nutriția în Embolia Pulmonară (PE-LNQ), versiunea 2.0

Protocol de studiu: Stil de viață, obiceiuri alimentare și factori de risc modificabili la pacienții cu embolie pulmonară acută (*Gib C., Anton A. et al.*)

Cod pacient: __________ Data completării: ____/____/________ Mod de completare: ☐ autocompletat ☐ asistat de investigator ☐ cu ajutorul aparținătorului Administrare: ☐ inițială ☐ re-test (7–14 zile)

Instrucțiuni pentru pacient. Acest chestionar face parte dintr-un studiu privind stilul de viață și alimentația persoanelor care au avut o embolie pulmonară. Vă rugăm să răspundeți gândindu-vă la obiceiurile dumneavoastră OBIȘNUITE din ultimele 12 luni DINAINTEA episodului actual, nu la perioada de spitalizare. Nu există răspunsuri corecte sau greșite. Bifați (☒) o singură căsuță, dacă nu se specifică altfel. Completarea durează aproximativ 15–20 de minute. Dacă nu sunteți sigur(ă) de un răspuns, alegeți varianta cea mai apropiată. Răspunsurile sunt confidențiale și sunt prelucrate doar sub cod.

A. Date sociodemografice și antropometrice

Sursa: itemi originali.

**A1**	Vârsta dumneavoastră:<br>________ ani
**A2**	Sexul:<br>☐ Feminin ☐ Masculin
**A3**	Mediul de rezidență:<br>☐ Urban ☐ Rural
**A4**	Ultimul nivel de studii absolvit:<br>☐ Fără studii / școală primară ☐ Gimnaziu ☐ Liceu / școală profesională ☐ Studii postliceale ☐ Studii universitare / postuniversitare
**A5**	Situația profesională și tipul activității (bifați una):<br>☐ Angajat(ă) – muncă predominant fizică ☐ Angajat(ă) – muncă predominant sedentară (la birou) ☐ Angajat(ă) – muncă mixtă ☐ Pensionar(ă) ☐ Fără ocupație / casnic(ă) ☐ Student(ă) ☐ Altele: ____________
**A6**	Înălțimea dumneavoastră:<br>________ cm
**A7**	Greutatea dumneavoastră obișnuită înainte de episodul actual:<br>________ kg ☐ Nu știu
**A8**	Cum s-a modificat greutatea dumneavoastră în ultimele 12 luni?<br>☐ Am crescut în greutate cu peste 5 kg ☐ Am scăzut în greutate cu peste 5 kg, intenționat (dietă, sport) ☐ Am scăzut în greutate cu peste 5 kg, neintenționat ☐ Greutate stabilă (±5 kg) ☐ Nu știu

B. Antecedente medicale și factori de risc pentru tromboembolism venos

Sursa: itemi originali. Răspunsurile vor fi confruntate cu foaia de observație.

**B1**	Ați mai avut înainte o tromboză venoasă profundă (cheag la picior) sau o embolie pulmonară?<br>☐ Nu ☐ Da – tromboză venoasă profundă, anul ______ ☐ Da – embolie pulmonară, anul ______
**B2**	Cineva din familia apropiată (părinți, frați/surori, copii) a avut tromboză venoasă sau embolie pulmonară?<br>☐ Nu ☐ Da ☐ Nu știu

**B3**	În ultimele 3 luni ați avut o operație, o fractură/traumatism major sau ați stat la pat mai mult de 3 zile?<br>☐ Nu ☐ Da – operație ☐ Da – fractură / traumatism ☐ Da – imobilizare la pat > 3 zile
**B4**	În ultimele 4 săptămâni ați făcut o călătorie de peste 4 ore fără întrerupere (mașină, autocar, tren, avion)?<br>☐ Nu ☐ Da
**B5**	Aveți în prezent sau ați avut în ultimii 5 ani o boală canceroasă sau tratament pentru cancer?<br>☐ Nu ☐ Da, în tratament activ ☐ Da, în urmărire / vindecat
**B6**	Urmați un tratament hormonal (anticoncepționale orale, tratament de substituție hormonală, alt tratament hormonal)?<br>☐ Nu ☐ Da: ____________________
**B7**	(Numai pentru femei) Sunteți însărcinată sau ați născut în ultimele 3 luni?<br>☐ Nu ☐ Da ☐ Nu se aplică
**B8**	Vi s-a spus vreodată că aveți o predispoziție la tromboze (trombofilie)?<br>☐ Nu ☐ Da ☐ Nu știu
**B9**	Ce boli cronice aveți diagnosticate? (bifați toate variantele valabile)<br>☐ Hipertensiune arterială ☐ Diabet zaharat ☐ Insuficiență cardiacă ☐ Boală pulmonară cronică (BPOC, astm) ☐ Boală renală cronică ☐ Varice / insuficiență venoasă ☐ Boală inflamatorie cronică (ex. poliartrită, boală inflamatorie intestinală) ☐ Colesterol crescut ☐ Niciuna ☐ Altele: ____________
**B10**	Ce medicamente luați în mod regulat? (bifați toate variantele valabile)<br>☐ Anticoagulant ☐ Antiagregant (aspirină, clopidogrel) ☐ Statină (pentru colesterol) ☐ Medicamente pentru diabet ☐ Medicamente pentru tensiune ☐ Niciunul ☐ Altele: ____________

C. Activitatea fizică (IPAQ-SF)

Sursa: International Physical Activity Questionnaire – Short Form (Craig et al., 2003), traducere românească realizată în cadrul studiului. Cadrul de referință: o săptămână obișnuită din ultimele 12 luni dinaintea episodului. Activitatea fizică VIGUROASĂ înseamnă efort intens, cu respirație mult accelerată; activitatea MODERATĂ înseamnă efort mediu, cu respirație ceva mai accelerată decât normal. Luați în calcul numai activitățile de cel puțin 10 minute fără întrerupere.

**C1**	Într-o săptămână obișnuită, în câte zile ați făcut activități fizice VIGUROASE (ex. ridicat greutăți, săpat, aerobic, mers rapid pe bicicletă)?<br>________ zile pe săptămână ☐ Nicio activitate viguroasă → treceți la C3
**C2**	Cât timp ați petrecut de obicei făcând activități fizice VIGUROASE într-una din acele zile?<br>________ ore ________ minute pe zi ☐ Nu știu / nu sunt sigur(ă)
**C3**	Într-o săptămână obișnuită, în câte zile ați făcut activități fizice MODERATE (ex. cărat greutăți ușoare, mers pe bicicletă în ritm normal, tenis de masă)? Nu includeți mersul pe jos.<br>________ zile pe săptămână ☐ Nicio activitate moderată → treceți la C5
**C4**	Cât timp ați petrecut de obicei făcând activități fizice MODERATE într-una din acele zile?<br>________ ore ________ minute pe zi ☐ Nu știu / nu sunt sigur(ă)
**C5**	Într-o săptămână obișnuită, în câte zile ați MERS PE JOS cel puțin 10 minute fără întrerupere (la serviciu, acasă, deplasări, plimbări)?<br>________ zile pe săptămână ☐ Deloc → treceți la C7
**C6**	Cât timp ați petrecut de obicei MERGÂND PE JOS într-una din acele zile?<br>________ ore ________ minute pe zi ☐ Nu știu / nu sunt sigur(ă)

**C7**	Într-o zi obișnuită din timpul săptămânii, cât timp ați petrecut ȘEZÂND (la birou, la masă, în fața televizorului sau calculatorului, în mașină)?<br>________ ore ________ minute pe zi ☐ Nu știu / nu sunt sigur(ă)

D. Aderența la dieta mediteraneană (MEDAS, 14 itemi)

Sursa: Mediterranean Diet Adherence Screener (Schröder et al., 2011; Martínez-González et al., 2012), traducere și adaptare românească realizate în cadrul studiului. O porție de legume = 200 g; o porție de carne = 100–150 g; o porție de nuci/semințe = 30 g; un pahar de vin = 100 ml.

**D1**	Folosiți uleiul de măsline ca principală grăsime pentru gătit?<br>☐ Da ☐ Nu
**D2**	Cât ulei de măsline consumați într-o zi (inclusiv cel folosit la prăjit, salate, mese în oraș)?<br>☐ 4 sau mai multe linguri ☐ Mai puțin de 4 linguri
**D3**	Câte porții de legume consumați pe zi? (1 porție = 200 g; garniturile se socotesc ca jumătate de porție)<br>☐ 2 sau mai multe (cel puțin una crudă sau ca salată) ☐ Mai puțin de 2
**D4**	Câte fructe (inclusiv sucuri naturale de fructe) consumați pe zi?<br>☐ 3 sau mai multe ☐ Mai puțin de 3
**D5**	Câte porții de carne roșie, hamburger sau produse din carne (șuncă, cârnați, salam, mezeluri) consumați pe zi? (1 porție = 100–150 g)<br>☐ Mai puțin de 1 ☐ 1 sau mai multe
**D6**	Câte porții de unt, margarină sau smântână consumați pe zi? (1 porție = 12 g)<br>☐ Mai puțin de 1 ☐ 1 sau mai multe
**D7**	Câte băuturi dulci sau carbogazoase (suc, cola, energizante) consumați pe zi?<br>☐ Mai puțin de 1 ☐ 1 sau mai multe
**D8**	Cât vin beți pe săptămână?<br>☐ 7 sau mai multe pahare ☐ Mai puțin de 7 pahare
**D9**	Câte porții de leguminoase (fasole, linte, mazăre, năut) consumați pe săptămână? (1 porție = 150 g)<br>☐ 3 sau mai multe ☐ Mai puțin de 3
**D10**	Câte porții de pește sau fructe de mare consumați pe săptămână? (1 porție = 100–150 g pește sau 200 g fructe de mare)<br>☐ 3 sau mai multe ☐ Mai puțin de 3
**D11**	De câte ori pe săptămână consumați dulciuri sau produse de patiserie/cofetărie din comerț (prăjituri, biscuiți, croasante, budinci)?<br>☐ Mai puțin de 3 ☐ 3 sau mai multe
**D12**	Câte porții de nuci, alune, migdale sau semințe consumați pe săptămână? (1 porție = 30 g)<br>☐ 3 sau mai multe ☐ Mai puțin de 3
**D13**	Preferați carnea de pui, curcan sau iepure în locul cărnii de vită, porc, hamburger sau cârnați?<br>☐ Da ☐ Nu
**D14**	De câte ori pe săptămână consumați legume, paste, orez sau alte mâncăruri gătite cu sos de roșii, ceapă, praz sau usturoi înăbușite în ulei de măsline (tip „sofrito")?<br>☐ 2 sau mai multe ☐ Mai puțin de 2

E. Obiceiuri alimentare complementare

Sursa: itemi originali.

**E1**	Câte mese principale luați de obicei pe zi?<br>☐ 1 ☐ 2 ☐ 3 ☐ Mai mult de 3 ☐ Neregulat, fără un program
**E2**	Cât de des luați micul dejun?<br>☐ Zilnic ☐ 4–6 zile pe săptămână ☐ 1–3 zile pe săptămână ☐ Rar sau niciodată
**E3**	Câtă apă (sau ceai neîndulcit) beți într-o zi obișnuită?<br>☐ Mai puțin de 1 litru ☐ 1–1,5 litri ☐ 1,5–2 litri ☐ Peste 2 litri
**E4**	Câte cești de cafea beți într-o zi obișnuită?<br>☐ Niciuna ☐ 1 ☐ 2–3 ☐ 4 sau mai multe
**E5**	În câte zile pe săptămână consumați alimente ultraprocesate (fast-food, snacks-uri sărate, mezeluri, produse de patiserie ambalate, mâncăruri semipreparate)?<br>☐ Niciodată / mai rar de 1 zi ☐ 1–2 zile ☐ 3–4 zile ☐ 5 sau mai multe zile
**E6**	Luați în mod regulat suplimente alimentare? (bifați toate variantele valabile)<br>☐ Nu ☐ Multivitamine / minerale ☐ Vitamina D ☐ Omega-3 / ulei de pește ☐ Proteine / suplimente pentru sport ☐ Plante medicinale / ceaiuri terapeutice ☐ Altele: ____________

F. Fumatul și dependența de nicotină

Sursa: itemi originali (F1–F5) și Fagerström Test for Nicotine Dependence (Heatherton et al., 1991; F6–F11), traducere românească realizată în cadrul studiului. Itemii F6–F11 se completează numai de fumătorii actuali.

**F1**	Care este situația dumneavoastră în privința fumatului (țigări)?<br>☐ Nu am fumat niciodată → treceți la F5 ☐ Fost fumător(oare) → completați F2–F5 ☐ Fumător(oare) actual(ă) → completați F2, F3 și F5–F11
**F2**	Câte țigări fumați (sau fumați) în medie pe zi?<br>________ țigări pe zi
**F3**	Câți ani ați fumat în total?<br>________ ani
**F4**	(Foști fumători) De câți ani/luni ați renunțat la fumat?<br>________ ani ________ luni
**F5**	Folosiți țigări electronice sau dispozitive cu tutun încălzit (ex. IQOS)?<br>☐ Nu ☐ Da, ocazional ☐ Da, zilnic ☐ Am folosit în trecut, acum nu
**F6**	(FTND 1) Cât de repede după ce vă treziți fumați prima țigară?<br>☐ În primele 5 minute (3) ☐ 6–30 de minute (2) ☐ 31–60 de minute (1) ☐ După mai mult de 60 de minute (0)
**F7**	(FTND 2) Vă este greu să nu fumați în locurile unde fumatul este interzis (biserică, bibliotecă, cinema, mijloace de transport)?<br>☐ Da (1) ☐ Nu (0)
**F8**	(FTND 3) La care țigară v-ar fi cel mai greu să renunțați?<br>☐ Prima de dimineață (1) ☐ Oricare alta (0)
**F9**	(FTND 4) Câte țigări fumați pe zi?<br>☐ 10 sau mai puține (0) ☐ 11–20 (1) ☐ 21–30 (2) ☐ 31 sau mai multe (3)
**F10**	(FTND 5) Fumați mai mult în primele ore după trezire decât în restul zilei?<br>☐ Da (1) ☐ Nu (0)

**F11**	(FTND 6) Fumați și atunci când sunteți atât de bolnav(ă) încât stați în pat mare parte din zi?<br>☐ Da (1) ☐ Nu (0)

G. Consumul de alcool

Sursa: itemi originali (format cantitate–frecvență). O unitate standard de alcool = 330 ml bere, 125 ml vin sau 40 ml băutură spirtoasă.

**G1**	Cât de des consumați băuturi alcoolice?<br>☐ Niciodată ☐ O dată pe lună sau mai rar ☐ 2–4 ori pe lună ☐ 2–3 ori pe săptămână ☐ 4 sau mai multe ori pe săptămână
**G2**	Într-o zi în care beți, câte unități standard de alcool consumați de obicei?<br>☐ 1–2 ☐ 3–4 ☐ 5–6 ☐ 7–9 ☐ 10 sau mai multe ☐ Nu se aplică (nu beau)
**G3**	Cât de des consumați 6 sau mai multe unități la o singură ocazie?<br>☐ Niciodată ☐ Mai rar de o dată pe lună ☐ Lunar ☐ Săptămânal ☐ Zilnic sau aproape zilnic

H. Percepția pacientului asupra evenimentului și intenția de schimbare

Sursa: itemi originali.

**H1**	Ce credeți că a contribuit la apariția emboliei pulmonare în cazul dumneavoastră? (bifați toate variantele valabile)<br>☐ Imobilizare / operație / călătorie lungă ☐ Greutatea corporală ☐ Lipsa mișcării ☐ Fumatul ☐ Alimentația ☐ Tratament hormonal ☐ O altă boală ☐ Nu știu ☐ Altceva: ____________________
**H2**	Înainte de acest episod, știați că greutatea, sedentarismul, fumatul și alimentația pot influența riscul de a face cheaguri de sânge?<br>☐ Da ☐ Parțial ☐ Nu
**H3**	Intenționați să vă schimbați în următoarele 6 luni unul dintre următoarele obiceiuri? (bifați toate variantele valabile)<br>☐ Să fac mai multă mișcare ☐ Să slăbesc ☐ Să renunț la fumat / să fumez mai puțin ☐ Să mănânc mai sănătos ☐ Să beau mai puțin alcool ☐ Nu intenționez schimbări ☐ Nu este cazul (nu am aceste obiceiuri)
**H4**	Ați dori să primiți consiliere sau sprijin (nutriționist, program de exerciții, consiliere pentru renunțarea la fumat) după externare?<br>☐ Da ☐ Nu ☐ Nu sunt sigur(ă) Observații: ____

Reguli de scorare (pentru investigator — nu se înmânează pacientului)

Secțiunea A. IMC pre-eveniment = greutate (A7, kg) / [înălțime (A6, m)]^2. Categorii OMS: subponderal < 18,5; normal 18,5–24,9; supraponderal 25,0–29,9; obezitate ≥ 30,0 kg/m^2. IMC-ul autoraportat se compară cu IMC-ul măsurat la internare (ipoteza de validitate v).

Secțiunea C (IPAQ-SF). MET-minute/săptămână = 8,0 × minute viguroase/zi × zile (C1–C2) + 4,0 × minute moderate/zi × zile (C3–C4) + 3,3 × minute mers/zi × zile (C5–C6). Valorile peste 180 min/zi pentru o activitate se trunchiază la 180 min. Categorii: RIDICATĂ = activitate viguroasă ≥ 3 zile cu ≥ 1500 MET-min/săpt. sau ≥ 7 zile de orice activitate cu ≥ 3000 MET-min/săpt.; MODERATĂ = ≥ 3 zile viguroase ≥ 20 min/zi, sau ≥ 5 zile moderate/mers ≥ 30 min/zi, sau ≥ 5 zile de orice combinație cu ≥ 600 MET-min/săpt.; SCĂZUTĂ = niciun criteriu îndeplinit. Timpul șezând (C7) se analizează separat, în minute/zi.

Secțiunea D (MEDAS). Se acordă 1 punct pentru fiecare item care îndeplinește criteriul (prima variantă listată la fiecare item) și 0 puncte în caz contrar. Scor total 0–14; ≥ 9 = aderență bună. Un singur item lipsă se scorează 0; scorul nu se calculează dacă lipsesc ≥ 2 itemi.

Secțiunea F. Pachete-ani = (țigări/zi [F2] ÷ 20) × ani de fumat [F3]. FTND (F6–F11): suma punctelor din paranteze, 0–10; 0–2 dependență foarte scăzută, 3–4 scăzută, 5 medie, 6–7 ridicată, 8–10 foarte ridicată. FTND se calculează numai dacă toți cei 6 itemi sunt completați.

Secțiunea G. Unități/săptămână = frecvența (G1: 0; 0,25; 0,75; 2,5; 4,5 zile/săpt.) × unități pe zi de consum (G2: punctul de mijloc al intervalului: 1,5; 3,5; 5,5; 8; 10). Categorii: abstinent; consum redus (< 7 unități/săpt. femei, < 14 bărbați); consum crescut (peste aceste praguri). G3 identifică consumul episodic excesiv.

Secțiunile B, E, H se raportează descriptiv (variabile categoriale); răspunsurile libere se codifică tematic.

PE-LNQ v2.0 poate fi utilizat gratuit în scopuri clinice și de cercetare necomerciale, cu citarea protocolului de studiu. Itemii IPAQ-SF, MEDAS și FTND aparțin autorilor instrumentelor originale.

Supplementary Material S2 — English translation of the pre-final Romanian patient-administered version, subject to the content validation and pilot testing described in the protocol

Pulmonary Embolism Lifestyle and Nutrition Questionnaire (PE-LNQ), version 2.0

Study protocol: Lifestyle, Dietary Habits, and Modifiable Risk Factors in Patients with Acute Pulmonary Embolism (Gib C., Anton A. *et al.*)

Patient code: __________ Date completed: ____/____/________ Mode: ☐ self-completed ☐ interviewer-assisted ☐ with proxy help Administration: ☐ baseline ☐ re-test (7–14 days)

Instructions for the patient. This questionnaire is part of a study on the lifestyle and diet of people who have had a pulmonary embolism. Please answer thinking of your USUAL habits during the 12 months BEFORE the current episode, not the period of hospitalization. There are no right or wrong answers. Tick (☒) one box unless stated otherwise. Completion takes about 15–20 minutes. If you are unsure, choose the closest answer. Your answers are confidential and are processed under a code only.

A. Sociodemographic and anthropometric data

Source: original items.

**A1**	Your age:<br>________ years
**A2**	Sex:<br>☐ Female ☐ Male
**A3**	Place of residence:<br>☐ Urban ☐ Rural
**A4**	Highest level of education completed:<br>☐ None / primary school ☐ Lower secondary ☐ High school / vocational ☐ Post-secondary ☐ University / postgraduate
**A5**	Employment status and type of work (tick one):<br>☐ Employed – mainly physical work ☐ Employed – mainly sedentary (desk) work ☐ Employed – mixed ☐ Retired ☐ Not employed / homemaker ☐ Student ☐ Other: ____________
**A6**	Your height:<br>________ cm
**A7**	Your usual weight before the current episode:<br>________ kg ☐ Don't know
**A8**	How did your weight change over the past 12 months?<br>☐ Gained more than 5 kg ☐ Lost more than 5 kg intentionally (diet, exercise) ☐ Lost more than 5 kg unintentionally ☐ Stable (±5 kg) ☐ Don't know

B. Medical history and venous thromboembolism risk factors

Source: original items. Answers are cross-checked against the medical record.

**B1**	Have you ever had a previous deep vein thrombosis (clot in the leg) or pulmonary embolism?<br>☐ No ☐ Yes – deep vein thrombosis, year ______ ☐ Yes – pulmonary embolism, year ______
**B2**	Has a first-degree relative (parents, siblings, children) had venous thrombosis or pulmonary embolism?<br>☐ No ☐ Yes ☐ Don't know

**B3**	In the past 3 months, did you have surgery, a fracture/major injury, or were you bed-bound for more than 3 days?<br>☐ No ☐ Yes – surgery ☐ Yes – fracture / injury ☐ Yes – bed rest > 3 days
**B4**	In the past 4 weeks, did you travel for more than 4 hours without a break (car, coach, train, plane)?<br>☐ No ☐ Yes
**B5**	Do you currently have, or have you had in the past 5 years, cancer or cancer treatment?<br>☐ No ☐ Yes, in active treatment ☐ Yes, in follow-up / cured
**B6**	Are you taking hormonal treatment (oral contraceptives, hormone replacement therapy, other hormonal treatment)?<br>☐ No ☐ Yes: ____________________
**B7**	(Women only) Are you pregnant or have you given birth in the past 3 months?<br>☐ No ☐ Yes ☐ Not applicable
**B8**	Have you ever been told that you have a clotting tendency (thrombophilia)?<br>☐ No ☐ Yes ☐ Don't know
**B9**	Which chronic conditions have you been diagnosed with? (tick all that apply)<br>☐ High blood pressure ☐ Diabetes ☐ Heart failure ☐ Chronic lung disease (COPD, asthma) ☐ Chronic kidney disease ☐ Varicose veins / venous insufficiency ☐ Chronic inflammatory disease (e.g., rheumatoid arthritis, inflammatory bowel disease) ☐ High cholesterol ☐ None ☐ Other: ____________
**B10**	Which medicines do you take regularly? (tick all that apply)<br>☐ Anticoagulant ☐ Antiplatelet (aspirin, clopidogrel) ☐ Statin (for cholesterol) ☐ Diabetes medicines ☐ Blood pressure medicines ☐ None ☐ Other: ____________

C. Physical activity (IPAQ-SF)

Source: International Physical Activity Questionnaire – Short Form (Craig et al., 2003); Romanian translation produced within the study. Reference frame: a usual week in the 12 months before the episode. VIGOROUS activity means hard physical effort that makes you breathe much harder than normal; MODERATE activity means moderate effort that makes you breathe somewhat harder than normal. Count only activities lasting at least 10 minutes at a time.

**C1**	In a usual week, on how many days did you do VIGOROUS physical activities (e.g., heavy lifting, digging, aerobics, fast bicycling)?<br>________ days per week ☐ No vigorous activity → skip to C3
**C2**	How much time did you usually spend doing VIGOROUS physical activities on one of those days?<br>________ hours ________ minutes per day ☐ Don't know / not sure
**C3**	In a usual week, on how many days did you do MODERATE physical activities (e.g., carrying light loads, bicycling at a regular pace, table tennis)? Do not include walking.<br>________ days per week ☐ No moderate activity → skip to C5
**C4**	How much time did you usually spend doing MODERATE physical activities on one of those days?<br>________ hours ________ minutes per day ☐ Don't know / not sure
**C5**	In a usual week, on how many days did you WALK for at least 10 minutes at a time (at work, at home, for travel, for recreation)?<br>________ days per week ☐ None → skip to C7

**C6**	How much time did you usually spend WALKING on one of those days?<br>________ hours ________ minutes per day ☐ Don't know / not sure
**C7**	On a usual weekday, how much time did you spend SITTING (at a desk, at the table, watching TV or using a computer, in a car)?<br>________ hours ________ minutes per day ☐ Don't know / not sure

D. Mediterranean diet adherence (MEDAS, 14 items)

Source: Mediterranean Diet Adherence Screener (Schröder et al., 2011; Martínez-González et al., 2012); Romanian translation and adaptation produced within the study. One serving of vegetables = 200 g; one serving of meat = 100–150 g; one serving of nuts = 30 g; one glass of wine = 100 ml.

**D1**	Do you use olive oil as the main culinary fat?<br>☐ Yes ☐ No
**D2**	How much olive oil do you consume in a given day (including that used for frying, salads, meals eaten away from home)?<br>☐ 4 or more tablespoons ☐ Fewer than 4 tablespoons
**D3**	How many vegetable servings do you consume per day? (1 serving = 200 g; side dishes count as half a serving)<br>☐ 2 or more (at least one raw or as salad) ☐ Fewer than 2
**D4**	How many fruit units (including natural fruit juices) do you consume per day?<br>☐ 3 or more ☐ Fewer than 3
**D5**	How many servings of red meat, hamburger, or meat products (ham, sausage, salami, cold cuts) do you consume per day? (1 serving = 100–150 g)<br>☐ Fewer than 1 ☐ 1 or more
**D6**	How many servings of butter, margarine, or cream do you consume per day? (1 serving = 12 g)<br>☐ Fewer than 1 ☐ 1 or more
**D7**	How many sweet or carbonated beverages (soft drinks, cola, energy drinks) do you consume per day?<br>☐ Fewer than 1 ☐ 1 or more
**D8**	How much wine do you drink per week?<br>☐ 7 or more glasses ☐ Fewer than 7 glasses
**D9**	How many servings of legumes (beans, lentils, peas, chickpeas) do you consume per week? (1 serving = 150 g)<br>☐ 3 or more ☐ Fewer than 3
**D10**	How many servings of fish or shellfish do you consume per week? (1 serving = 100–150 g fish or 200 g shellfish)<br>☐ 3 or more ☐ Fewer than 3
**D11**	How many times per week do you consume commercial sweets or pastries (cakes, cookies, croissants, custard)?<br>☐ Fewer than 3 ☐ 3 or more
**D12**	How many servings of nuts (including peanuts, almonds) or seeds do you consume per week? (1 serving = 30 g)<br>☐ 3 or more ☐ Fewer than 3
**D13**	Do you prefer chicken, turkey, or rabbit meat instead of veal, pork, hamburger, or sausage?<br>☐ Yes ☐ No

**D14**	How many times per week do you consume vegetables, pasta, rice, or other dishes seasoned with a sauce of tomato, onion, leek, or garlic simmered in olive oil (sofrito)?<br>☐ 2 or more ☐ Fewer than 2

E. Complementary dietary habits

Source: original items.

**E1**	How many main meals do you usually eat per day?<br>☐ 1 ☐ 2 ☐ 3 ☐ More than 3 ☐ Irregular, no set pattern
**E2**	How often do you eat breakfast?<br>☐ Every day ☐ 4–6 days a week ☐ 1–3 days a week ☐ Rarely or never
**E3**	How much water (or unsweetened tea) do you drink on a usual day?<br>☐ Less than 1 liter ☐ 1–1.5 liters ☐ 1.5–2 liters ☐ More than 2 liters
**E4**	How many cups of coffee do you drink on a usual day?<br>☐ None ☐ 1 ☐ 2–3 ☐ 4 or more
**E5**	On how many days per week do you eat ultra-processed foods (fast food, salty snacks, processed meats, packaged pastries, ready meals)?<br>☐ Never / less than 1 day ☐ 1–2 days ☐ 3–4 days ☐ 5 or more days
**E6**	Do you regularly take dietary supplements? (tick all that apply)<br>☐ No ☐ Multivitamins / minerals ☐ Vitamin D ☐ Omega-3 / fish oil ☐ Protein / sports supplements ☐ Herbal products ☐ Other: ____________

F. Smoking and nicotine dependence

Source: original items (F1–F5) and the Fagerström Test for Nicotine Dependence (Heatherton et al., 1991; F6–F11); Romanian translation produced within the study. Items F6–F11 are completed by current smokers only.

**F1**	What is your cigarette smoking status?<br>☐ Never smoked → skip to F5 ☐ Former smoker → complete F2–F5 ☐ Current smoker → complete F2, F3 and F5–F11
**F2**	How many cigarettes do (did) you smoke per day on average?<br>________ cigarettes per day
**F3**	For how many years have you smoked in total?<br>________ years
**F4**	(Former smokers) How long ago did you quit smoking?<br>________ years ________ months
**F5**	Do you use electronic cigarettes or heated-tobacco devices (e.g., IQOS)?<br>☐ No ☐ Yes, occasionally ☐ Yes, daily ☐ Used in the past, not now
**F6**	(FTND 1) How soon after you wake up do you smoke your first cigarette?<br>☐ Within 5 minutes (3) ☐ 6–30 minutes (2) ☐ 31–60 minutes (1) ☐ After 60 minutes (0)
**F7**	(FTND 2) Do you find it difficult to refrain from smoking in places where it is forbidden (church, library, cinema, public transport)?<br>☐ Yes (1) ☐ No (0)
**F8**	(FTND 3) Which cigarette would you most hate to give up?<br>☐ The first one in the morning (1) ☐ Any other (0)

**F9**	(FTND 4) How many cigarettes per day do you smoke?<br>☐ 10 or fewer (0) ☐ 11–20 (1) ☐ 21–30 (2) ☐ 31 or more (3)
**F10**	(FTND 5) Do you smoke more frequently during the first hours after waking than during the rest of the day?<br>☐ Yes (1) ☐ No (0)
**F11**	(FTND 6) Do you smoke even if you are so ill that you are in bed most of the day?<br>☐ Yes (1) ☐ No (0)

G. Alcohol intake

Source: original items (quantity–frequency format). One standard drink = 330 ml beer, 125 ml wine, or 40 ml spirits.

**G1**	How often do you drink alcoholic beverages?<br>☐ Never ☐ Monthly or less ☐ 2–4 times a month ☐ 2–3 times a week ☐ 4 or more times a week
**G2**	On a day when you drink, how many standard drinks do you usually have?<br>☐ 1–2 ☐ 3–4 ☐ 5–6 ☐ 7–9 ☐ 10 or more ☐ Not applicable (I do not drink)
**G3**	How often do you have 6 or more drinks on one occasion?<br>☐ Never ☐ Less than monthly ☐ Monthly ☐ Weekly ☐ Daily or almost daily

H. Patient perception of the event and intentions to change

Source: original items.

**H1**	What do you think contributed to your pulmonary embolism? (tick all that apply)<br>☐ Immobility / surgery / long travel ☐ Body weight ☐ Lack of physical activity ☐ Smoking ☐ Diet ☐ Hormonal treatment ☐ Another illness ☐ Don't know ☐ Other: ____________________
**H2**	Before this episode, did you know that body weight, inactivity, smoking, and diet can influence the risk of blood clots?<br>☐ Yes ☐ Partly ☐ No
**H3**	In the next 6 months, do you intend to change any of the following? (tick all that apply)<br>☐ Be more physically active ☐ Lose weight ☐ Quit / cut down smoking ☐ Eat more healthily ☐ Drink less alcohol ☐ No changes intended ☐ Not applicable (I do not have these habits)
**H4**	Would you like to receive counselling or support (dietitian, exercise program, smoking-cessation counselling) after discharge?<br>☐ Yes ☐ No ☐ Not sure Comments: ____

Scoring rules (for the investigator — not handed to the patient)

Section A. Pre-event BMI = weight (A7, kg) / [height (A6, m)]^2. WHO categories: underweight < 18.5; normal 18.5–24.9; overweight 25.0–29.9; obesity ≥ 30.0 kg/m^2. Self-reported BMI is compared with measured admission BMI (validity hypothesis v).

Section C (IPAQ-SF). MET-minutes/week = 8.0 × vigorous minutes/day × days (C1–C2) + 4.0 × moderate minutes/day × days (C3–C4) + 3.3 × walking minutes/day × days (C5–C6). Values above 180 min/day for any activity are truncated to 180 min. Categories: HIGH = vigorous activity on ≥ 3 days reaching ≥ 1500 MET-min/week, or ≥ 7 days of any combination reaching ≥ 3000 MET-min/week; MODERATE = ≥ 3 days of vigorous activity ≥ 20 min/day, or ≥ 5 days of moderate activity/walking ≥ 30 min/day, or ≥ 5 days of any combination reaching ≥ 600 MET-min/week; LOW = no criterion met. Sitting time (C7) is analyzed separately, in minutes/day.

Section D (MEDAS). One point is awarded for each item meeting the criterion (the first option listed for each item) and 0 otherwise. Total score 0–14; ≥ 9 = good adherence. A single missing item is scored 0; the score is not computed if ≥ 2 items are missing.

Section F. Pack-years = (cigarettes/day [F2] ÷ 20) × years smoked [F3]. FTND (F6–F11): sum of the bracketed points, 0–10; 0–2 very low, 3–4 low, 5 medium, 6–7 high, 8–10 very high dependence. FTND is computed only if all 6 items are completed.

Section G. Units/week = frequency (G1: 0; 0.25; 0.75; 2.5; 4.5 days/week) × drinks per drinking day (G2: interval midpoint: 1.5; 3.5; 5.5; 8; 10). Categories: abstinent; low intake (< 7 units/week women, < 14 men); higher intake (above these thresholds). G3 identifies heavy episodic drinking.

Sections B, E, and H are reported descriptively (categorical variables); free-text answers are coded thematically.

PE-LNQ v2.0 may be used freely for non-commercial clinical and research purposes with citation of the study protocol. The IPAQ-SF, MEDAS and FTND items belong to the authors of the original instruments.

Material suplimentar S3 — Fișa de informare a participantului și Formularul de consimțământ informat (versiunea în limba română, administrată pacientului)

FIȘA DE INFORMARE A PARTICIPANTULUI ȘI CONSIMȚĂMÂNT INFORMAT

Studiul: „Stil de viață, obiceiuri alimentare și factori de risc modificabili la pacienții cu embolie pulmonară acută: studiu observațional prospectiv cu dezvoltarea și validarea unui chestionar compozit de stil de viață și nutriție (PE-LNQ)"

Instituția: Spitalul Clinic Municipal de Urgență Timișoara, Secția Clinică de Medicină Internă (aviz instituțional nr. E-3780/18.08.2026), în cadrul Școlii Doctorale a Universității de Medicină și Farmacie „Victor Babeș" din Timișoara.

Investigator principal: Dr. Carina Gib, medic rezident Medicină Internă, doctorand. Coordonator: Prof. univ. dr. Daniel Florin Lighezan, șef Secție Clinică de Medicină Internă.

Contact pentru întrebări legate de studiu: Dr. Carina Gib, carina.gib@umft.ro; Secția Clinică de Medicină Internă, B-dul Regele Mihai I nr. 19, Timișoara.

1. Invitație și scopul studiului

Sunteți invitat(ă) să participați la un studiu de cercetare medicală deoarece ați fost internat(ă) cu diagnosticul de embolie pulmonară (cheag de sânge ajuns în vasele plămânilor). Studiul urmărește să înțeleagă dacă obiceiurile de viață dinaintea îmbolnăvirii — greutatea corporală, activitatea fizică, alimentația, fumatul și consumul de alcool — au legătură cu gravitatea emboliei pulmonare și cu evoluția din primele luni după episod. Studiul dezvoltă și verifică, totodată, un chestionar (PE-LNQ) care să poată fi folosit ulterior de medici pentru evaluarea acestor obiceiuri. Vă rugăm să citiți cu atenție aceste informații și să puneți orice întrebare înainte de a decide. Decizia de a participa vă aparține în întregime.

2. Ce presupune participarea

Dacă acceptați, vi se va cere: (a) să completați un chestionar despre obiceiurile dumneavoastră din ultimele 12 luni dinaintea îmbolnăvirii (aproximativ 15–20 de minute; puteți fi ajutat(ă) de un membru al echipei sau de un aparținător); (b) eventual, să completați din nou același chestionar după 7–14 zile, pentru a verifica dacă răspunsurile sunt stabile în timp — acest lucru va fi solicitat numai unei părți dintre participanți; (c) să permiteți măsurarea circumferinței taliei cu un centimetru (singura măsurătoare adăugată de studiu); (d) să permiteți echipei de cercetare să consulte din foaia dumneavoastră de observație datele clinice și analizele de sânge efectuate în mod obișnuit la internare (nu se recoltează nicio probă suplimentară pentru studiu); (e) să fiți contactat(ă) telefonic sau la controlul programat, la 3 și la 6 luni de la externare, pentru câteva întrebări despre starea dumneavoastră de sănătate. Studiul nu modifică în niciun fel tratamentul sau îngrijirea pe care le primiți.

3. Riscuri și disconfort

Participarea nu implică riscuri medicale. Singurele neajunsuri sunt timpul necesar completării chestionarului și posibilul disconfort de a răspunde la întrebări despre greutate, fumat sau alcool. Puteți sări peste orice întrebare la care nu doriți să răspundeți.

4. Beneficii

Este posibil să nu aveți un beneficiu direct. Discuția despre obiceiurile de viață poate fi însă un prilej de a primi sfaturi pentru prevenirea unui nou episod. Rezultatele studiului vor ajuta medicii să înțeleagă mai bine rolul stilului de viață în embolia pulmonară și vor pune la dispoziția comunității medicale un instrument validat de evaluare.

5. Participare voluntară și retragere

Participarea este voluntară. Puteți refuza sau vă puteți retrage din studiu în orice moment, fără a da explicații și fără nicio consecință asupra îngrijirii medicale pe care o primiți acum sau în viitor. În cazul retragerii, datele colectate până în acel moment vor fi șterse la cererea dumneavoastră, cu excepția celor deja incluse în analize anonimizate.

6. Confidențialitate și protecția datelor

Datele dumneavoastră vor fi prelucrate în conformitate cu Regulamentul (UE) 2016/679 (GDPR) și cu legislația română în vigoare. Chestionarul și datele clinice vor fi înregistrate sub un cod numeric, fără nume sau alte date de

identificare; lista de corespondență dintre cod și identitate este păstrată separat, într-un loc securizat, accesibil numai investigatorului principal și coordonatorului, și va fi distrusă la încheierea studiului. Datele codificate vor fi stocate pe suport securizat pentru o perioadă de 10 ani de la finalizarea studiului, apoi vor fi distruse. Rezultatele vor fi publicate în reviste medicale și prezentate la conferințe numai sub formă agregată, fără posibilitatea identificării vreunui participant. Aveți dreptul de acces, rectificare, ștergere, restricționare a prelucrării și portabilitate a datelor, precum și dreptul de a depune o plângere la Autoritatea Națională de Supraveghere a Prelucrării Datelor cu Caracter Personal. Operatorul de date este Spitalul Clinic Municipal de Urgență Timișoara (nr. operator 37058); responsabilul cu protecția datelor poate fi contactat prin secretariatul spitalului (secretariat@smtm.ro).

7. Costuri și compensații

Participarea nu implică niciun cost pentru dumneavoastră și nu se acordă compensații financiare. Studiul nu beneficiază de finanțare externă.

8. Întrebări

Pentru orice întrebare despre studiu sau despre drepturile dumneavoastră ca participant, vă puteți adresa investigatorului principal (datele de contact de mai sus) sau conducerii Secției Clinice de Medicină Internă.

CONSIMȚĂMÂNT INFORMAT (se completează de participant)

☐ Am citit (sau mi-a fost citită) fișa de informare de mai sus, am înțeles scopul studiului și ce presupune participarea și am avut posibilitatea de a pune întrebări, la care am primit răspunsuri satisfăcătoare.

☐ Înțeleg că participarea este voluntară și că mă pot retrage oricând, fără explicații și fără consecințe asupra îngrijirii mele medicale.

☐ Sunt de acord să completez chestionarul PE-LNQ și, dacă mi se va solicita, să îl completez din nou după 7–14 zile.

☐ Sunt de acord cu măsurarea circumferinței taliei.

☐ Sunt de acord ca echipa de cercetare să consulte din foaia mea de observație datele clinice și rezultatele analizelor efectuate în mod obișnuit la internare.

☐ Sunt de acord să fiu contactat(ă) la 3 și la 6 luni de la externare, telefonic sau la control, pentru urmărirea evoluției. Număr de telefon: ________________________

☐ Sunt de acord ca datele mele, codificate, să fie prelucrate și păstrate conform celor descrise la punctul 6 și ca rezultatele să fie publicate în formă agregată.

☐ Sunt de acord să particip la acest studiu.

Participant — Nume și prenume (litere de tipar): ______________________________ Semnătura: ________________ Data: ____/____/________

Reprezentant legal / martor (numai dacă participantul nu poate citi sau semna; se completează în prezența participantului, cu acordul verbal al acestuia) — Nume și prenume: ____________________________ Calitatea: _____________ Semnătura: _______________ Data: ____/____/________

Investigator care a obținut consimțământul — Declar că am explicat participantului natura, scopul și procedurile studiului și că am răspuns la întrebările acestuia. Nume și prenume: ______________________________ Semnătura: ________________ Data: ____/____/________

Formularul se semnează în două exemplare originale: unul se înmânează participantului, celălalt se păstrează în dosarul studiului. Cod participant atribuit: ___________

Supplementary Material S3 — Participant Information Sheet and Informed Consent Form (English translation of the Romanian patient-administered version)

PARTICIPANT INFORMATION SHEET AND INFORMED CONSENT FORM

Study: "Lifestyle, Dietary Habits, and Modifiable Risk Factors in Patients with Acute Pulmonary Embolism: a prospective observational study with development and validation of a composite lifestyle and nutrition questionnaire (PE-LNQ)"

Institution: Municipal Emergency Clinical Hospital of Timișoara, Clinical Department of Internal Medicine (institutional approval No. E-3780/18.08.2026), within the Doctoral School of the "Victor Babeș" University of Medicine and Pharmacy Timișoara.

Principal investigator: Dr. Carina Gib, resident in Internal Medicine, doctoral student. Coordinator: Prof. Daniel Florin Lighezan, MD, PhD, Head of the Clinical Department of Internal Medicine.

Contact for questions about the study: Dr. Carina Gib, carina.gib@umft.ro; Clinical Department of Internal Medicine, 19 Regele Mihai I Blvd., Timișoara.

1. Invitation and purpose of the study

You are invited to take part in a medical research study because you have been admitted with a diagnosis of pulmonary embolism (a blood clot that has reached the vessels of the lungs). The study aims to understand whether lifestyle habits before the illness — body weight, physical activity, diet, smoking, and alcohol use — are related to the severity of pulmonary embolism and to its course in the first months after the episode. The study also develops and tests a questionnaire (PE-LNQ) that doctors may later use to assess these habits. Please read this information carefully and ask any questions before deciding. The decision to take part is entirely yours.

2. What participation involves

If you agree, you will be asked: (a) to complete a questionnaire about your habits during the 12 months before the illness (about 15–20 minutes; a member of the study team or a relative may help you); (b) possibly, to complete the same questionnaire again after 7–14 days to check whether the answers are stable over time — this will be requested from only some participants; (c) to allow a tape measurement of your waist circumference (the only measurement added by the study); (d) to allow the research team to consult, from your medical record, the clinical data and the blood tests routinely performed at admission (no additional sample is taken for the study); (e) to be contacted by telephone or at the scheduled visit, at 3 and 6 months after discharge, for a few questions about your health. The study does not change in any way the treatment or care you receive.

3. Risks and discomfort

Participation involves no medical risk. The only inconveniences are the time needed to complete the questionnaire and possible discomfort in answering questions about weight, smoking, or alcohol. You may skip any question you do not wish to answer.

4. Benefits

You may not receive a direct benefit. However, discussing lifestyle habits may be an opportunity to receive advice on preventing a new episode. The results will help doctors better understand the role of lifestyle in pulmonary embolism and will provide the medical community with a validated assessment tool.

5. Voluntary participation and withdrawal

Participation is voluntary. You may refuse or withdraw from the study at any time, without giving reasons and without any consequence for the medical care you receive now or in the future. If you withdraw, the data collected up to that point will be deleted at your request, except for data already included in anonymized analyses.

6. Confidentiality and data protection

Your data will be processed in accordance with Regulation (EU) 2016/679 (GDPR) and Romanian legislation in force. The questionnaire and clinical data will be recorded under a numerical code, without your name or other identifying data; the list linking the code to your identity is kept separately, in a secure location accessible only to the principal investigator and the coordinator, and will be destroyed at the end of the study. Coded data will be

stored on secure media for 10 years after completion of the study and then destroyed. Results will be published in medical journals and presented at conferences only in aggregated form, without the possibility of identifying any participant. You have the right to access, rectify, erase, restrict the processing of, and port your data, and the right to lodge a complaint with the National Supervisory Authority for Personal Data Processing. The data controller is the Municipal Emergency Clinical Hospital of Timișoara (operator No. 37058); the data protection officer can be contacted through the hospital secretariat (secretariat@smtm.ro).

7. Costs and compensation

Participation involves no cost to you and no financial compensation is provided. The study receives no external funding.

8. Questions

For any question about the study or about your rights as a participant, you may contact the principal investigator (contact details above) or the management of the Clinical Department of Internal Medicine.

INFORMED CONSENT (to be completed by the participant)

☐ I have read (or have had read to me) the information sheet above, I understand the purpose of the study and what participation involves, and I have had the opportunity to ask questions, which have been answered satisfactorily.

☐ I understand that participation is voluntary and that I may withdraw at any time, without giving reasons and without consequences for my medical care.

☐ I agree to complete the PE-LNQ questionnaire and, if asked, to complete it again after 7–14 days.

☐ I agree to the measurement of my waist circumference.

☐ I agree that the research team may consult, from my medical record, the clinical data and the results of the tests routinely performed at admission.

☐ I agree to be contacted at 3 and 6 months after discharge, by telephone or at the follow-up visit. Telephone number: ________________________

☐ I agree that my coded data may be processed and stored as described in section 6 and that the results may be published in aggregated form.

☐ I agree to take part in this study.

Participant — Full name (block capitals): ______________________________ Signature: ________________ Date: ____/____/________

Legal representative / witness (only if the participant cannot read or sign; completed in the participant's presence, with the participant's verbal agreement) — Full name: ____________________________ Relationship: _____________ Signature: _______________ Date: ____/____/________

Investigator obtaining consent — I confirm that I have explained the nature, purpose, and procedures of the study to the participant and have answered their questions. Full name: _____________________________ Signature: ________________ Date: ____/____/________

The form is signed in two original copies: one is given to the participant, the other is kept in the study file. Participant code assigned: ____________

Table S4. STROBE Statement — checklist of items for reports of cohort studies, completed for the present protocol.

Reference: von Elm E, et al. Lancet 2007;370:1453–1457 [Ref. 71]. Because this is a study protocol, items pertaining to results (13–17) cannot be completed; the section of the protocol in which each item is planned is indicated, and the results publication will complete them.

Section	Item	Recommendation	Location in the protocol
Title and abstract	1	(a) Indicate the study's design with a commonly used term in the title or the abstract	Title ("prospective observational study"); Abstract
		(b) Provide in the abstract an informative and balanced summary of what was done and what was found	Abstract (Background/Objectives, Methods, Expected Results, Conclusions)
Introduction	2	Background/rationale: explain the scientific background and rationale for the investigation	Section 1, paragraphs 1–8
	3	Objectives: state specific objectives, including any pre-specified hypotheses	Section 1, final paragraph (two components, hypotheses and endpoints)
Methods	4	Study design: present key elements of study design early in the paper	Section 2.1
	5	Setting: describe the setting, locations, and relevant dates, including periods of recruitment, exposure, follow-up, and data collection	Section 2.1 (center; criteria for additional centers); Section 2.6 (follow-up at discharge, 3 and 6 months)
	6	(a) Participants: give the eligibility criteria, and the sources and methods of selection of participants; describe methods of follow-up	Section 2.2 (eligibility, consecutive enrollment); Section 2.6 (follow-up)
		(b) For matched studies, give matching criteria and number of exposed and unexposed	Not applicable (no matching)
	7	Variables: clearly define all outcomes, exposures, predictors, potential confounders, and effect modifiers; give diagnostic criteria, if applicable	Section 2.4.1 and Table 2 (clinical/laboratory variables, covariates); Section 2.4.2 and Table 3 (exposures); Section 2.4.3 (outcomes); Section 2.7.2 (adjustment set)
	8	Data sources/measurement: for each variable of interest, give sources of data and details of methods of assessment; describe comparability of assessment methods if there is more than one group	Section 2.4.1 (medical record, laboratory information system, timing of sampling); Section 2.4.2 and Section 2.5 (PE-LNQ); Section 2.6 (standardized administration)
	9	Bias: describe any efforts to address potential sources of bias	Section 2.8 (selection, recall, information bias); Section 2.6 (standardized questionnaire timing and assistance)
	10	Study size: explain how the study size was arrived at	Section 2.3 and Table 1 (scenario grid, staged targets, EPV budget)
	11	Quantitative variables: explain how quantitative variables were handled in the analyses; describe which groupings were chosen and why	Section 2.7.1 (distribution, log-transformation); Section 2.7.2 (categorical and continuous exposure coding); Section 2.4.1 (age-adjusted D-dimer, CONUT, metabolic syndrome)
	12	(a) Statistical methods: describe all statistical methods, including those used to control for confounding	Section 2.7.1–2.7.4; Supplementary Methods S6
		(b) Describe any methods used to examine subgroups and interactions	Section 2.7.4 and S6 (diet × smoking interaction; subgroups in Section 2.7.6)
		(c) Explain how missing data were addressed	Section 2.7.5 (complete case; multiple imputation)
		(d) If applicable, explain how loss to follow-up was addressed	Section 2.3 (15% attrition allowance); Section 2.7.3 (time-to-event with censoring; competing risks)

Section	Item	Recommendation	Location in the protocol
		(e) Describe any sensitivity analyses	Sections 2.4.3, 2.7.2, 2.7.4, 2.7.5; S6
Results	13	Participants: report numbers at each stage; reasons for non-participation; flow diagram	Not applicable to the protocol — will be reported in the results publication (STROBE flow diagram planned)
	14	Descriptive data: characteristics of participants, missing data, follow-up time	Not applicable — results publication
	15	Outcome data: report numbers of outcome events or summary measures over time	Not applicable — results publication
	16	Main results: unadjusted and adjusted estimates with precision; category boundaries; absolute risk	Not applicable — results publication (planned in Sections 2.7.2–2.7.3: 95% CI, absolute risks)
	17	Other analyses: subgroups, interactions, sensitivity analyses	Not applicable — results publication (planned in Section 2.7.4 and S6)
Discussion	18	Key results: summarise key results with reference to study objectives	Section 3 (Expected Results)
	19	Limitations: discuss limitations, taking into account sources of potential bias or imprecision	Section 4, final paragraph
	20	Interpretation: give a cautious overall interpretation considering objectives, limitations, multiplicity of analyses, and other relevant evidence	Sections 3 and 4; analysis hierarchy in Section 2.7.1
	21	Generalisability: discuss the external validity of the study results	Section 4 (single-center first stage, multicenter extension, validation in a single population)
Other information	22	Funding: give the source of funding and the role of the funders	Funding statement (no external funding)

Table S5. COSMIN reporting guideline for studies on measurement properties of patient-reported outcome measures (version 2.0), mapped to the sections of the present protocol.

Reference: Gagnier JJ, de Arruda GT, Terwee CB, Mokkink LB. Qual Life Res 2025;34:1901–1911 [Ref. 70]. Items on results and their interpretation will be completed in the validation publication; the section in which each item is planned is indicated.

Domain	Reporting item (summarized)	Location in the protocol
Title	Identify the study as an evaluation of measurement properties, naming the instrument	Title ("Development and Validation of a Composite Lifestyle and Nutrition Questionnaire")
Abstract	Structured summary: instrument, population, measurement properties assessed, methods, (expected) results	Abstract (Phase 1 endpoints)
Introduction	Background and rationale for evaluating the instrument in this population; constructs measured	Section 1, paragraphs 7–8
	Objectives and hypotheses of the measurement study	Section 1, final paragraph (measurement component)
Methods — design	Study design and setting of the measurement study	Section 2.1 (Phase 1); Section 2.5
Methods — population	Target population, inclusion/exclusion criteria, recruitment, sampling	Section 2.2 (validation subsamples drawn from the cohort; test–retest stability criteria)
	Sample-size justification for each measurement property	Section 2.3, "Validation phase" (≥ 8 experts; 15 pilot patients; ≥ 130 for psychometrics; ≥ 80 for test–retest)
Methods — instrument	Description of the PROM: construct(s), items, subscales, scoring, recall period, administration mode	Section 2.4.2 and Table 3; Questionnaires S1–S2 (scoring rules)
	Development and translation/cross-cultural adaptation procedure	Section 2.5.1 (forward–backward translation, expert committee)
	Administration: timing, mode, assistance	Section 2.6 (standardized timing, self-completion, interviewer assistance, proxy rules, mode recorded)
Methods — measurement properties	Content validity: procedure, raters, criteria	Section 2.5.2 (expert panel, I-CVI/S-CVI thresholds); Section 2.5.3 (cognitive debriefing, acceptability)
	Structural validity	Not assessed: constituent scales retain their validated structure; MEDAS is a formative index (Section 2.5.4)
	Internal consistency	Sections 2.5.4 and 2.7.6 (Cronbach's alpha for FTND; descriptive for MEDAS)
	Reliability (test–retest): interval, stability criteria, statistic	Sections 2.5.4, 2.6 and 2.7.6 (7–14 days; ICC(2,1); weighted and Cohen's kappa)
	Measurement error	Section 2.7.6 (SEM, MDC95; Bland–Altman limits of agreement)
	Criterion validity	Not applicable (no gold standard); self-reported vs. measured BMI treated as agreement (Section 2.5.4, hypothesis v)
	Hypotheses testing for construct validity: a priori hypotheses, expected direction and magnitude, comparators	Section 2.5.4 (hypotheses i–v with expected |r|; objective clinical and laboratory anchors); Section 2.7.6 (≥ 75% confirmation criterion)
	Cross-cultural validity / measurement invariance	Section 2.5.1 (adaptation process); Section 2.7.6 (stability across age, sex, administration mode — descriptive)
	Responsiveness	Not assessed (single pre-event lifestyle assessment)
	Interpretability: floor/ceiling effects, score	Section 2.7.6 (15% criterion)

Domain	Reporting item (summarized)	Location in the protocol
	distributions	
Methods — statistics	Statistical methods for each property, software, handling of missing items	Section 2.7.1 (software); Section 2.7.5 (item-level missingness rules); Section 2.7.6
Methods — ethics	Ethical approval and consent	Section 2.9; IRB and Informed Consent statements; Consent Form S3
Results	Sample characteristics; results per measurement property with precision; comparison with hypotheses	To be reported in the validation publication
Discussion	Interpretation of measurement properties; limitations; generalizability; recommendations for use	Sections 3 (expected) and 4 (limitations: single population; formative MEDAS)
Other information	Availability of the instrument and scoring rules; funding	Data Availability Statement; Questionnaires S1–S2; Funding statement

Supplementary Methods S6. Detailed Specification of Secondary and Exploratory Analyses

Supplement to: Lifestyle, Dietary Habits, and Modifiable Risk Factors in Patients with Acute Pulmonary Embolism: Protocol for a Prospective Observational Study with Development and Validation of a Composite Lifestyle and Nutrition Questionnaire. Reference numbers correspond to the reference list of the main article.

All analyses below are secondary, supportive, or exploratory according to the hierarchy defined in Section 2.7.1 of the main text. Exploratory analyses are hypothesis-generating and will be reported with effect estimates and 95% confidence intervals irrespective of statistical significance.

S6.1. Key Secondary Analysis and Severity Sensitivity Analyses

The ordinal PESI class (I–V) will be modeled with proportional-odds regression using the exposures and adjustment set of the primary analysis; the proportional-odds assumption will be verified with the Brant test, and a partial proportional-odds model will be used if it is violated. In sensitivity analyses, (i) the four-level 2019 ESC classification (low, intermediate-low, intermediate-high, and high risk), derived from hemodynamic status, PESI/sPESI, CTPA RV/LV ratio, and troponin [3], will be modeled as an ordinal outcome; and (ii) the primary logistic analysis will be repeated after exclusion of high-risk (hemodynamically unstable) patients.

S6.2. Time-to-Event Analyses

The early adverse-outcome composite will be analyzed as a binary endpoint (in-hospital) and as a time-to-event endpoint (3 and 6 months) with Kaplan–Meier curves, the log-rank test, and Cox proportional-hazards models; proportionality will be verified with Schoenfeld residuals. For recurrent VTE, a Fine–Gray subdistribution-hazards model treating death as a competing event will be fitted as a sensitivity analysis. Given the expected number of events (Section 2.3), Cox models in the single-center stage will be parsimonious (exposure plus one to two covariates at EPV ≥ 10).

S6.3. Incremental Prognostic Value (Exploratory)

The incremental prognostic value of lifestyle factors will be quantified against two reference models—the sPESI alone, and the sPESI plus the Tier 1 biomarkers troponin and lactate—as the change in area under the ROC curve (DeLong test), supplemented by the continuous net reclassification improvement and integrated discrimination improvement, interpreted with the caution appropriate to these metrics. Absolute risks and risk differences will accompany all relative measures.

S6.4. Biomarker Correlations, Sensitivity Adjustment, Mediation, Dose–Response, and Interaction (Exploratory)

Correlations between lifestyle indicators and continuous severity markers (RV/LV ratio, troponin, natriuretic peptides, D-dimer, lactate) and Tier 2–3 laboratory parameters will use Pearson or Spearman coefficients, with partial correlations adjusting for age and sex and Benjamini–Hochberg false-discovery-rate control across the family of tests. D-dimer will be log-transformed and analyzed with age in the model; age-adjusted D-dimer positivity (age × 10 µg/L above 50 years) will be reported descriptively [75–77].

In sensitivity analyses, the primary logistic model will be additionally adjusted for sodium, eGFR, glucose, albumin, and CRP to examine whether lifestyle–severity associations are independent of, or attenuated by, these routine markers. An exploratory mediation analysis (product-of-coefficients method with bias-corrected bootstrap confidence intervals) will quantify the proportion of the association between lifestyle exposures and severity transmitted through cardiometabolic and inflammatory markers.

Potential non-linear dose–response relationships for MET-minutes/week and MEDAS score will be explored with restricted cubic splines (three knots at the 10th, 50th, and 90th percentiles). A pre-specified

multiplicative interaction between Mediterranean-diet adherence and smoking status will be tested, motivated by prior evidence of effect modification [32].

S6.5. Nutritional Status: CONUT Score (Exploratory)

The Controlling Nutritional Status (CONUT) score will be computed from the first routine sample as the sum of three components: serum albumin ($\geq$ 3.5 g/dL = 0; 3.0–3.49 = 2; 2.5–2.99 = 4; < 2.5 = 6 points), total cholesterol ($\geq$ 180 mg/dL = 0; 140–179 = 1; 100–139 = 2; < 100 = 3 points), and total lymphocyte count ($\geq$ 1600/μL = 0; 1200–1599 = 1; 800–1199 = 2; < 800 = 3 points), yielding 0–12 points (0–1 normal; 2–4 mild; 5–8 moderate; 9–12 severe undernutrition). The score will be analyzed continuously and dichotomized at > 4, the threshold associated with in-hospital mortality in acute PE [52], in relation to severity class and early adverse outcomes, with the adjustment set of the primary analysis. Because albumin and cholesterol are influenced by the acute-phase response and cholesterol by lipid-lowering therapy [82], models will be adjusted for CRP and for lipid-lowering therapy.

S6.6. LDL Cholesterol and Bleeding During Anticoagulation (Exploratory)

Bleeding events during follow-up will be classified as ISTH major bleeding [80] or clinically relevant non-major bleeding. The association between admission LDL cholesterol (< 70 vs. $\geq$ 70 mg/dL, i.e., < 1.8 vs. $\geq$ 1.8 mmol/L, as in the RIETE analysis [54]; and continuously) and time to first bleeding event will be examined with Fine–Gray models treating death as a competing event, adjusted parsimoniously for age, renal function (eGFR), anemia, active cancer, and antiplatelet therapy, with statin therapy recorded as a potential effect modifier. Given the expected small number of bleeding events, particularly in the single-center stage, results will be reported primarily descriptively (event counts and cumulative incidences by LDL category).
